# The active E4 structure of nitrogenase studied with different DFT functionals

**DOI:** 10.1002/jcc.26435

**Published:** 2020-10-14

**Authors:** Wen‐Jie Wei, Per E. M. Siegbahn

**Affiliations:** ^1^ Key Laboratory of Material Chemistry for Energy Conversion and Storage, Ministry of Education, Hubei Key Laboratory of Bioinorganic Chemistry and Materia Medica, Hubei Key Laboratory of Materials Chemistry and Service Failure, School of Chemistry and Chemical Engineering Huazhong University of Science and Technology Wuhan China; ^2^ Department of Organic Chemistry, Arrhenius Laboratory Stockholm University Stockholm Sweden

**Keywords:** density functional theory, energetics, nitrogenase, the E4 state

## Abstract

The present study concerns the technical aspects of obtaining the energetics for the E4 state of nitrogenase, the enzyme that fixes N_2_ in nature. EPR experiments have shown that the critical E4 structure that activates N_2_ should contain two bridging hydrides in the FeMo‐cofactor. It is furthermore in equilibrium with a structure where the two hydrides have been released and N_2_ binds. These observations led to the suggestion that E4 should have two bridging hydrides and two protonated sulfides. It is important to note that the structure for E4 has not been determined, but only suggested. For a long time, no DFT study led to the suggested structure, independent of which functional was used. However, in two recent DFT studies a good agreement with the experimental suggestion was claimed to have been obtained. In one of them the TPSS functional was used. That was the first out of 11 functionals tried that led to the experimentally suggested structure. In the second of the recent DFT studies, a similar conclusion was reached using the TPSSh functional. The conclusions in the recent studies have here been studied in detail, by calculating a critical energetic value strongly implied by the same EPR experiments. Both the TPSS and TPSSh functionals have been used. The present calculations suggest that those DFT functionals would not lead to agreement with the experimental EPR results either.

## Graphical abstract

Nitrogenase is the enzyme in nature that fixes nitrogen from the air. The structure of the ground state of the enzyme has been known for decades, but the mechanism for how the enzyme works has remained elusive. The structure for the active state where N2 binds has been suggested based on EPR. It is shown in the present study that DFT, independent of functional used, cannot reproduce the experimental suggestion.

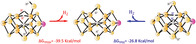

## 1 INTRODUCTION

**FIGURE 1:**
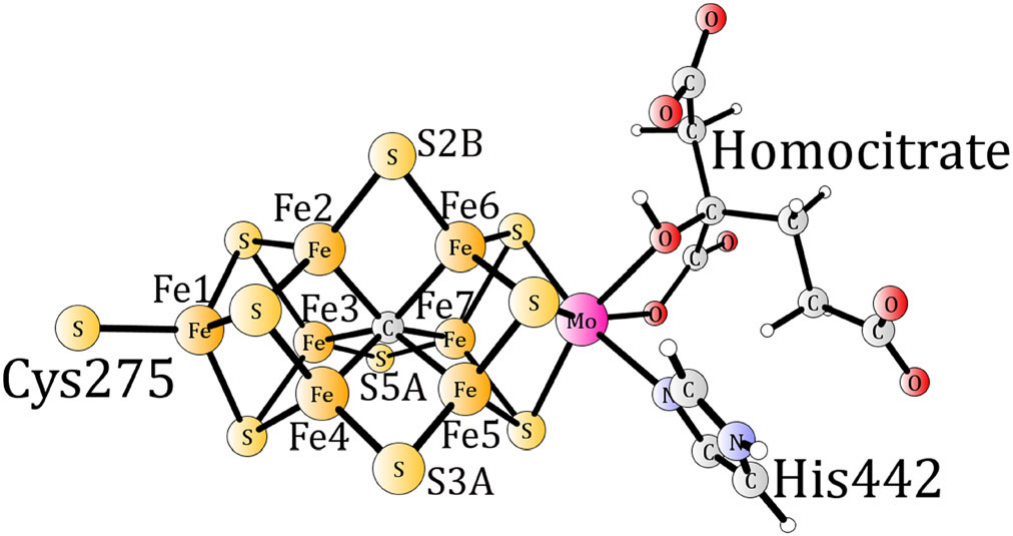
X‐ray structure for the MoFe‐cofactor of nitrogenase [Color figure can be viewed at wileyonlinelibrary.com]

Nitrogenase is the enzyme in nature that fixes nitrogen from the air and produces ammonia. This is a key step for making use of nitrogen, for example, in the synthesis of amino acids that are the building blocks of the proteins. The active site of nitrogenase is quite complicated.^1^ It has seven irons and one molybdenum connected by sulfide bonds, see Figure 1. There are variants of this complex, where vanadium or iron replaces molybdenum, but they are much less common.

The present study was motivated by two recent theoretical papers. In one of these, Ryde et al.^2^ got results suggesting that the TPSS functional^3^ does reproduce the experimental findings for E4, the key species in N_2_ activation by nitrogenase. In the other one, Björnsson et al^4^ showed that the TPSSh functional^5^ might also work. The suggested E4 structures were obtained after only four reductions. In our previous studies, we have not been able to reproduce the experimentally obtained energetics for E4, if the structure should be obtained after only four reductions, as suggested experimentally. Instead, four additional reductions were required to find an E4 structure that agreed with the experimental findings concerning both structure and energetics. The additional reductions were suggested to occur only once before catalysis starts. Our previous studies strongly indicated that, in fact, no DFT functional would reproduce the energetics after only four reductions.

Another previous study by Ryde et al gives very important background information for the present study.^6^ It was shown that after trying 10 different DFT functionals, none of them reproduced the experimentally suggested E4 structure. The energy for the experimentally suggested structure was shown to always be much higher in energy than other structures for all functionals.

The focus in the present study is on the technical aspects of theoretical treatments of the nitrogenase mechanism. Still, a short background on the most relevant experimental and theoretical work is given here. The mechanism for binding nitrogen, has turned out to be very challenging. In a series of important papers, Hoffman, Seefeldt and coworkers have been able to capture the E4 active structure, the one that binds nitrogen.^7^, ^8^ The EPR analysis showed that E4 has two bridging hydrides which leave as a hydrogen molecule, and allows nitrogen to bind in an altogether reversible step. They showed that the loss of H_2_ is obligatory for binding N_2_, which explains a long‐lasting mystery that one H_2_ is formed for each N_2_ molecule bound. The E4 state was suggested to be formed after four initial reductions in the catalytic cycle, in agreement with the Lowe‐Thorneley basic findings for nitrogenase.^9^, ^10^ Since E4 is obtained after four reductions in the catalytic cycling and found to have two hydrides, the remaining two hydrogens were suggested to bind to the sulfides. There is one very surprising conclusion from the suggestion of the active E4 structure. With two hydrides and two protonated sulfides, the oxidation states of the metals are the same as for the initial ground state before reduction. The oxidation state should therefore contain four Fe (III), and after hydride loss there will still be two Fe (III). Even though the reductant has a very low redox potential of −1.6 V, the lowest in nature, the oxidation states of the metals in E4 are therefore quite high, and should not be particularly electron‐donating, as was expected for the most difficult reduction in nature. There was one other finding, very important in the present context, showing that after catalysis the cofactor goes back to its initial ground state. This has meant, that so far the only structure that has been structurally characterized by X‐ray crystallography is the ground state, termed E0. The structures of the reduced E‐states, including E4, have just been indirectly assumed.

The experimentally deduced structure for the active E4 state, became controversial after a series of theoretical studies.^11^, ^12^, ^13^ The suggested E4 structure was found to be very high in energy using the B3LYP functional (for both 15% or 20% exact exchange). The experimental E4 structure, with a central carbide and two hydrides, was shown to be more than 30 kcal/mol higher than the lowest energy structure, which was found to have a triply protonated carbide. Still, it is in principle possible that B3LYP completely fails in this particular case in a way not seen for any other enzyme. Therefore, tests were made by changing the fraction of exact exchange, which has been shown to be the most sensitive parameter in B3LYP.^14^ The most critical aspect for testing this parameter is for the step where the two hydrides leave to form H_2_ in the step where N_2_ becomes bound. That step was well established experimentally to be reversible, suggesting a small energy difference between the reactant and final E4 states. The DFT tests for this step showed that the loss of H_2_ is very exergonic, by more than 40 kcal/mol, independent of the fraction of exact exchange, which was varied between 0% and 20%. This result suggests that all DFT functionals should have a similar problem for the suggested E4 structure. In the two recent theoretical studies by Ryde et al^2^ and Björnsson et al^4^ of the E4 state, the energetics of that step was not investigated.

A clarification is needed concerning a recent dismissal^15^ of the mechanism suggested by the present calculations.^11^, ^12^, ^13^, ^14^ That dismissal is incorrect, since the number of electrons in the suggested E4 structure has been misread. The criticism was that the theoretically suggested structure would lead to a singlet state for E4, since it should have an even number of electrons, which would be in disagreement with the EPR analysis which shows that E4 is a doublet state. However, it is clearly stated in the theoretical paper that the suggested E4 structure has an odd number of electrons in agreement with EPR.

## 2 COMPUTATIONAL DETAILS

The present study only concerns calculations using TPSS^3^ and TPSSh.^5^ For the calculations with the TPSSh functional, (model 1, Figure S1) is the same as the one used previously for nitrogenase, consisting of about 270 atoms.^11^, ^12^, ^13^, ^14^ For the calculations with the TPSS functional, two extra water molecules are added into the previous model, as suggested by the study by Ryde et al (model2, Figure S2). Some backbone atoms were kept fixed from the X‐ray structure, as shown in detail in the SI. The basis set used for the geometry optimization was the LACVP* basis set. For the optimized geometries, single point calculations were performed with a large basis set with cc‐pvtz(−f) for all atoms except for the metals, which were described by the LACV3P+ basis. Dielectric effects^16^ with a dielectric constant of 4.0 and the D2 dispersion effects^17^ were also added as usual. This is the standard procedure used for a large number of enzyme mechanisms.^18^ Translational entropy is important in the present study. For the loss of H_2,_ there is a gain of translational entropy of 8.4 kcal/mol, and for the binding of N_2,_ there is a loss of 9.9 kcal/mol. Both calculated from the particle‐in‐a box‐scheme. The calculations were performed using the Jaguar^16^ and Gaussian^19^ programs.

## 3 RESULTS

The present study concerns the suggested structures of the E4 state as obtained after four reductions from the experimentally well characterized ground state E0. Only the TPSS^3^

and TPSSh^5^ functionals have been used here. For comparison the energetic results for E4 obtained in the previous B3LYP study^14^ are given in Table 1. For the nomenclature used, (C, 2H^−^) has a central carbon and two hydrides; (CH_3_) has a terminal CH_3_ and no hydrides; (C‐H_2_) has a central carbide and a free H_2_. The percentages given refer to the amount of exact exchange used in the B3LYP functional. The two rows of highest interest here are the ones of (C, 2H^−^) and (C‐H_2_). (C, 2H^−^) gives the energy for the experimentally suggested structure, here set to zero, and (C‐H_2_) the energy of the structure, for which H_2_ has been removed. It should first be noted that for all fractions of exact exchange, the energies are much lower for (C‐H_2_), showing that the removal of the two hydrides is very exergonic. The exergonicity for 0% (non‐hybrid) is 34.0 kcal/mol, for 10% it is 43.3 kcal/mol, for 15% it is 48.0 kcal/mol and for 20% it is 54.6 kcal/mol. Since the fraction of exact exchange is by far the most sensitive parameter in B3LYP, the results suggest that the large exergonicity would remain for all DFT functionals. The experimental analysis requires that the loss of H_2_ together with the binding of N_2_ should be almost thermoneutral, since it is easily reversible. A thermoneutral E4 step and a very exergonic H_2_ release, would require a very strong binding of N_2_, which is contrary to what is found in any calculation so far, particularly, when the loss of translational entropy of 9.9 kcal/mol is accounted for. Instead a very weak binding is found. Therefore, the calculated results show that all the B3LYP functionals predict a very exergonic E4 step for the experimentally suggested structure, in conflict with the experimental EPR analysis. The conclusion was therefore that the experimentally suggested structure is not correct. There has been one theoretical study suggesting how this problem might be solved for the experimentally suggested structure.^20^ To allow the disappearance of the hydrides and still prevent the energy loss going to a free H_2_, the molecularly bound H_2_ state formed was suggested to be prevented from being released from the cofactor by very high barriers. These barriers have to be at least 20 kcal/mol, but this was not demonstrated in the suggested mechanism. In a later study,^13^ only a small barrier of a few kcal/mol was found for releasing H_2_, which would not be enough to explain the experiments.

**TABLE 1:** Relative energies (kcal/mol) for the E4 state of nitrogenase, using B3LYP density functionals with different fractions of exact exchange.^14^ TPSS and TPSSh functionals

Structure		B3LYP			TPSS	TPSSh
	0%	10%	15%	20%		
C,2H^−^	0	0	0	0	0	0
CH_3_	−6.1	−38.9	−62.6	−81.8		
C‐H_2_	−34.0	−43.3	−48.0	−54.6	−26.8	−39.5

**FIGURE 2:**
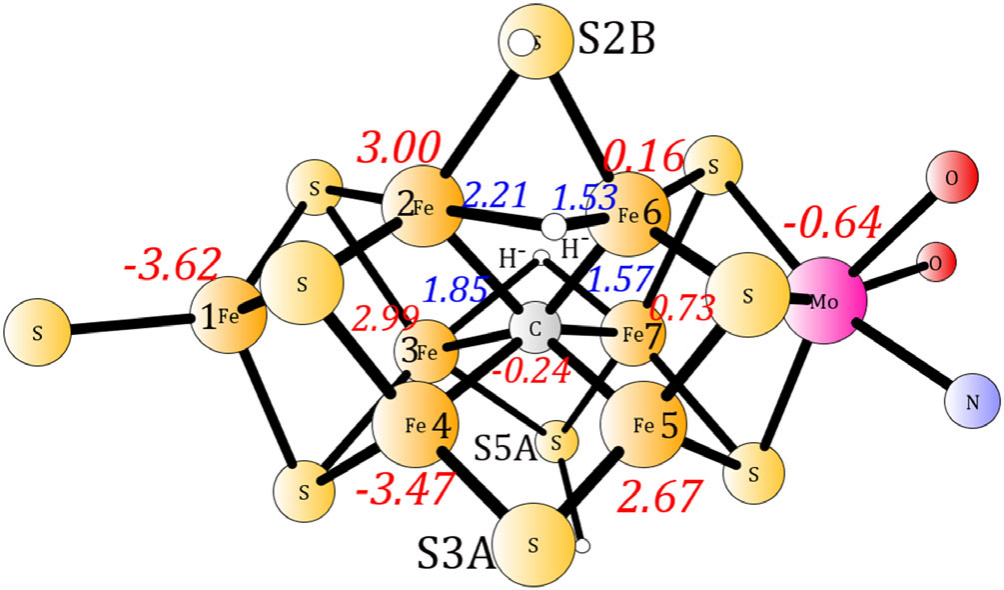
The TPSS optimized structure (C, 2H^−^) with two bridging hydrides and a central carbide. Distances (Å) between the iron and the hydrides are shown in blue italic. Spin populations are shown in red italic. For clarity, only the core of the model is shown [Color figure can be viewed at wileyonlinelibrary.com]

Even considering the results described above, there have been two recent suggestions that there should be functionals that still reproduce the experimental energetics. Ryde et al showed that using TPSS,^2^ the lowest energy E4 structure is indeed the experimentally suggested structure, with a central carbide and two bridging hydrides, which was therefore suggested to reproduce the experimentally suggested mechanism. That would be the first DFT functional that gave that result. In light of the previous results in Table 1, this would be very surprising. In order to investigate if the suggestion by Ryde et al is possible, the TPSS functional was used for calculating the energy difference between the (C, 2H^−^) and the (C‐H_2_) structures. The optimized structure for (C, 2H^−^) is shown in Figure 2. The spin‐distribution, including the alternations of the spins, is the same as in the Ryde et al study, and the spin‐populations are very similar. The positions of the hydrides are also the same. Removing H_2_ from the cofactor to reach the (C‐H_2_) structure, keeping the same sulfides protonated, reduces the energy by −16.6 kcal/mol. The optimized structure is shown in Figure S3. For the (C‐H_2_) structure, when moving the proton from S5A to S3A, the energy goes down by an additional −10.2 kcal/mol, which means that the exergonicity drops to −26.8 kcal/mol. The optimized structure is shown in Figure S4. For the entries in Table 1, 0% has a difference of −34.0 kcal/mol, and should be the one most similar to the non‐hybrid TPSS method. It should be noted that slightly different models have been used. It is clear that the E4 structure suggested by TPSS, is not able to give reasonable energetics for the nitrogenase mechanism, even though the structure with two hydrides agrees with the one suggested for E4 by experiments.

**FIGURE 3:**
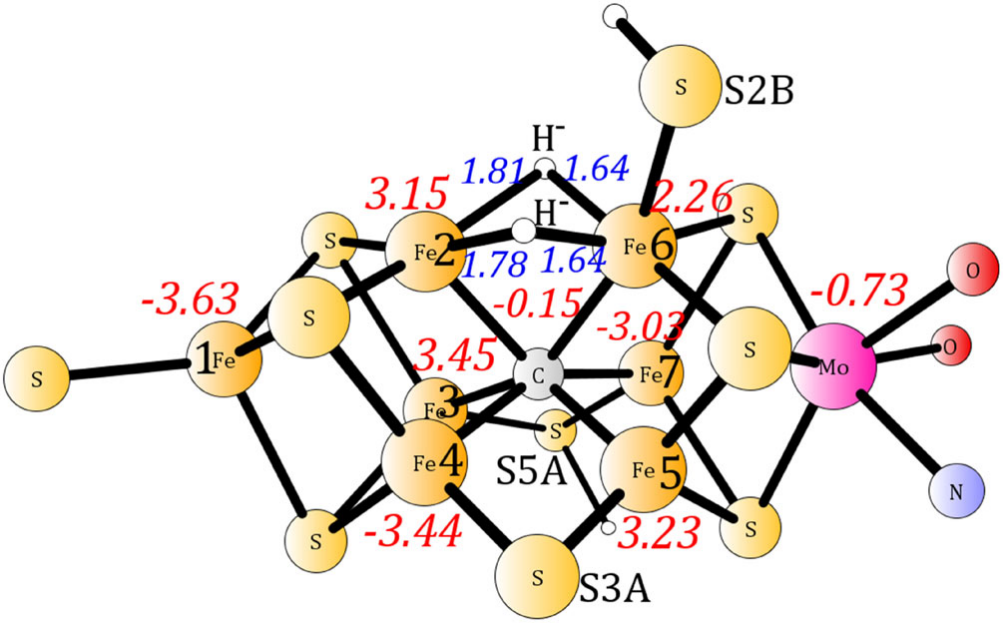
The TPSSh optimized structure (C, 2H^−^) with two hydrides and a central carbide. Distances (Å) between the iron and the hydrides are shown in blue italic. Mulliken spin densities are shown in red italic. For clarity, only the core of the model is shown [Color figure can be viewed at wileyonlinelibrary.com]

In the second recent DFT study by Björnsson et al^4^ discussed here, the hybrid method TPSSh^5^ was used. The energetics of the critical part in the E4 mechanism was not investigated in spite of the results demonstrated earlier.^14^ The conclusion obtained in that study was that the experimentally suggested E4 structure would lead to reasonable energetics for the nitrogenase mechanism. In order to test their suggestion, the E4 energetics was calculated using TPSSh. The spin distribution and structure was chosen to be the same as in the previous TPSSh study. The spin‐populations are very similar. The resulting structure with two hydrides is shown in Figure 3, where also the spin‐populations are given. The hydrides are bound to the same irons as in the previous study. There are several di‐hydride structures with similar energies, as found also in the previous study. This finding indicates that moving the hydrides around should not require high barriers. With the methods used here, the dihydride structure (Figure S5) in our previous study is −0.4 kcal/mol lower than the one in Figure 3. The energetics for removing the hydrides as a hydrogen molecule was then calculated. As in the comparison above to the previous TPSS study, the release of H_2_ was again found to be very exergonic, now by −32.6 kcal/mol. The optimized structure is shown in Figure S6. Also, when moving the proton from S5A to S3A, the energy decreases by another −6.9 kcal/mol, leading to an exergonicity of −39.5 kcal/mol. The optimized structure is shown in Figure S7.

## 4 CONCLUSIONS

The two recent suggestions, that TPSS^2^ or TPSSh^4^ could be used for calculating the energetics of the nitrogenase mechanism, have been investigated by calculating the most critical part of the N_2_ activation in nitrogenase. In previous studies by Ryde et al, 10 different DFT functionals were tried and none of them reproduced the experimentally suggested structure with two hydrides and two protonated sulfides, actually very from it. In a later study by Ryde et al, it was shown that the TPSS functional, as the first DFT functional, gave the suggested structure. The presence of two bridging hydrides in E4 has been shown by experiments, beyond doubt. It has also been shown that the hydrides should leave as H_2_ in a reversible step as N_2_ binds. This indicates that the release of H_2_ should be almost thermoneutral. In contrast, in the present study, it has been shown that the TPSS functional gives an exergonicity of −26.8 kcal/mol, and the TPSSh functional one of −39.5 kcal/mol. These results are in line with a previous study, systematically investigating this energy difference by varying the fraction of exact exchange.^14^ This means that all DFT functionals investigated so far, agree on the finding that the experimentally suggested structure leads to energetics of the E4 state, which is very far from the conclusions drawn by the analysis of the EPR experiments.

In the above context, it is important to point out that a different scenario has been suggested by theoretical modeling, which leads to both a structure and energetics in very good agreement with the analysis of the EPR experiments.^11^, ^12^, ^13^, ^14^ The difference to the experimentally suggested mechanism is that an additional four reduction steps are introduced before catalysis starts. These reduction steps lead to an oxidation state for the cofactor without any Fe(III) present in E4. After hydride loss as H_2_, there will actually be two Fe(I) atoms in the structure, making it very reducing for the activation of N_2_. The suggested mechanism is also in full agreement with the Lowe‐Thorneley analysis, since the activation step occurs only once before the reductions in the actual catalytic cycle starts. As previously shown, also the energetics of the remaining protonation steps to reach the NH_3_ product are in good agreement with available experimental thermodynamics and kinetics. At the end of the catalytic cycling, the cofactor is very highly excited due to the reductions and will eventually fall down into its lowest energy state (E0), as all systems in nature do. The lowest energy state is the initial state before reduction, which is therefore the state observed also after catalysis.

There are two possible conclusions that can be drawn from the present study. The first one is that DFT should not be used to study nitrogenase, since there are errors much larger than seen for any other similar enzyme. The second possibility is that the experimentally suggested structure is not correct. An alternative structure has been suggested with a protonated carbide with good agreement to the experimentally deduced structure and energetics.^13^

## Supporting information


**Appendix** **S1**: Supporting Information
