# Supplementary material for: The active E4 structure of nitrogenase studied with different DFT functionals
Source: J Comput Chem. 2020 Oct 14;42(2):81–5. doi: 10.1002/jcc.26435 (PMC7756797; doi:10.1002/jcc.26435)
Supplement: Supplementary file 1 — Appendix S1: Supporting Information [file JCC-42-81-s001.docx]

***Supporting Information for***

**The active E4 structure of nitrogenase studied with different DFT functionals**

Wen-Jie Wei,^*a^ Per E. M. Siegbahn^*b^

^a^Key Laboratory of Material Chemistry for Energy Conversion and Storage, Ministry of Education, Hubei Key Laboratory of Bioinorganic Chemistry and Materia Medica, Hubei Key Laboratory of Materials Chemistry and Service Failure, School of Chemistry and Chemical Engineering, Huazhong University of Science and Technology, Wuhan 430074, China

^b^Department of Organic Chemistry, Arrhenius Laboratory, Stockholm University, SE-106 91, Stockholm, Sweden

^*^Corresponding authors: [wen-jie.wei@su.se](mailto:wen-jie.wei@su.se) ; [per.siegbahn@su.se](mailto:per.siegbahn@su.se)


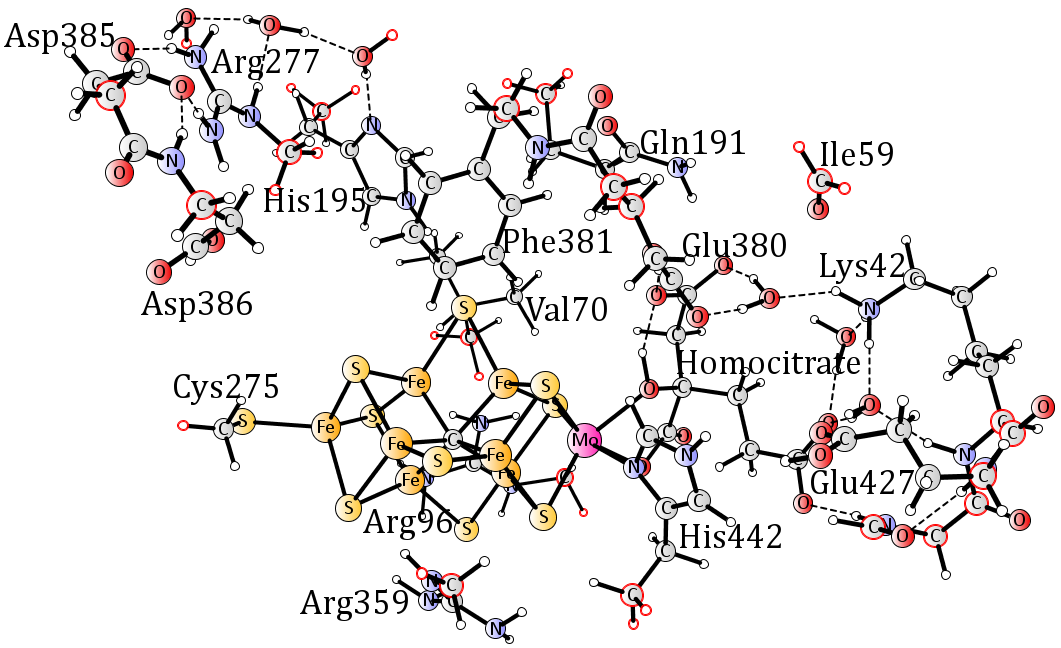


***Figure S1****. Structure of the model 1. Atoms marked with red circles were fixed at their X-ray structure positions during the geometry optimizations.*

The fixed atom’s numbers are as follows:

33, 34, 70, 84, 85, 86, 99, 100, 101, 113, 114, 127, 131 142, 159, 160, 161, 163, 164, 165, 166, 178, 183, 196, 207, 225, 230, 233, 247, 248, 249, 250, 254, 269, 270, 271


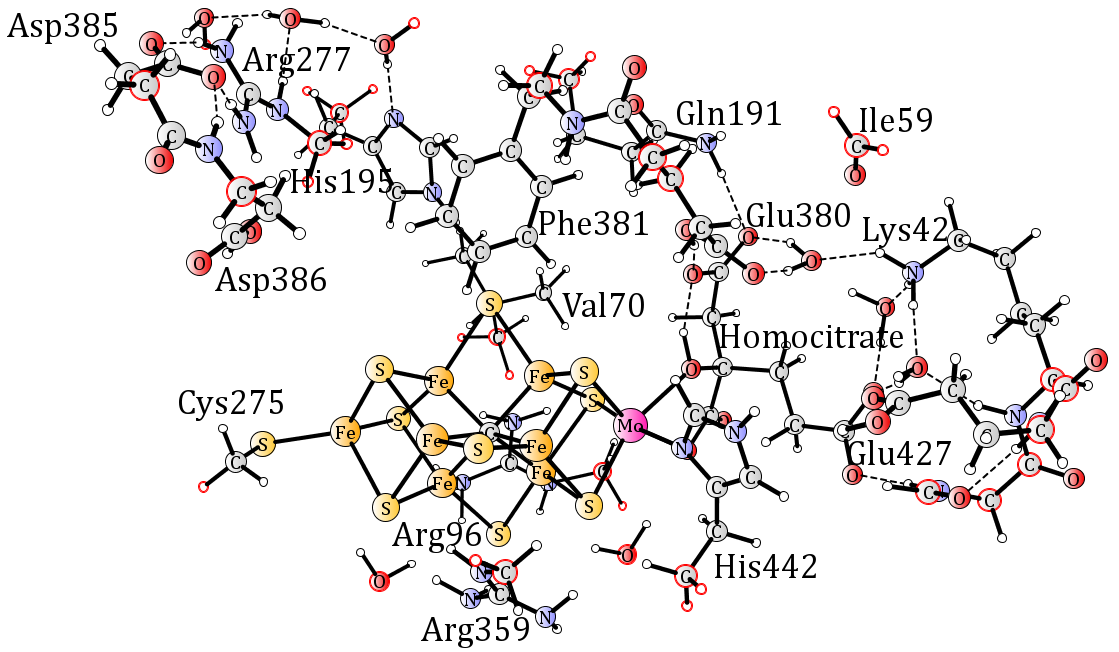


***Figure S2.*** *Structure of the model 2. Atoms marked with red circles were fixed at their X-ray structure positions during the geometry optimizations.*

The fixed atom’s numbers are as follows:

33, 34, 70, 84, 85, 86, 99, 100, 101, 113, 114, 127, 131 142, 159, 160, 161, 163, 164, 165, 166, 178, 183, 196, 207, 225, 230, 233, 247, 248, 249, 250, 254, 269, 270, 271, 273, 274


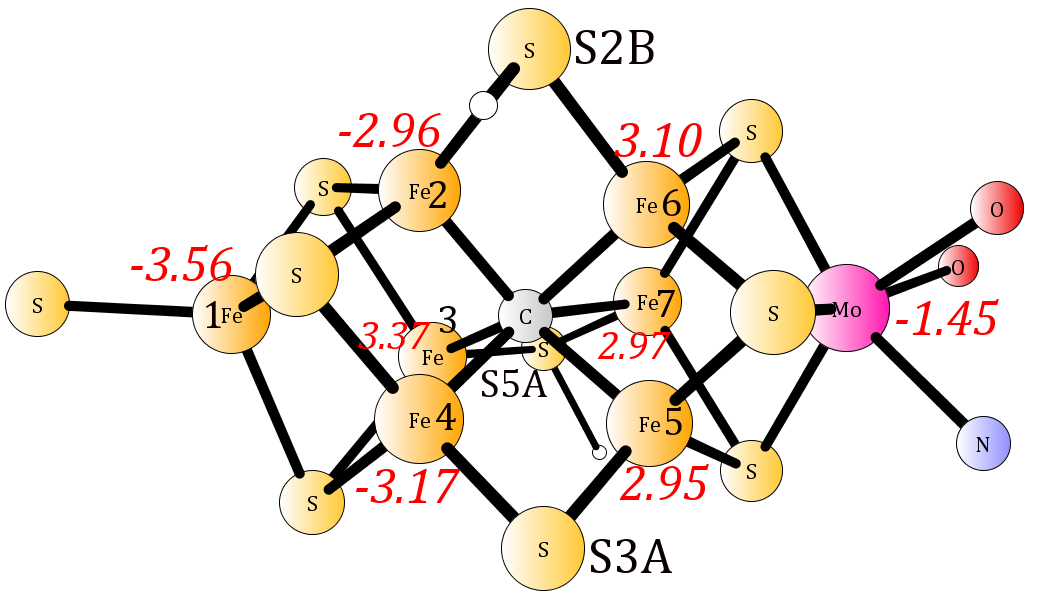


***Figure S3.*** *The TPSS optimized structure (C-H2), protons are on the S2B and S5A. Mulliken spin densities are shown in red italic. For clarity, only the core of the model is shown.*


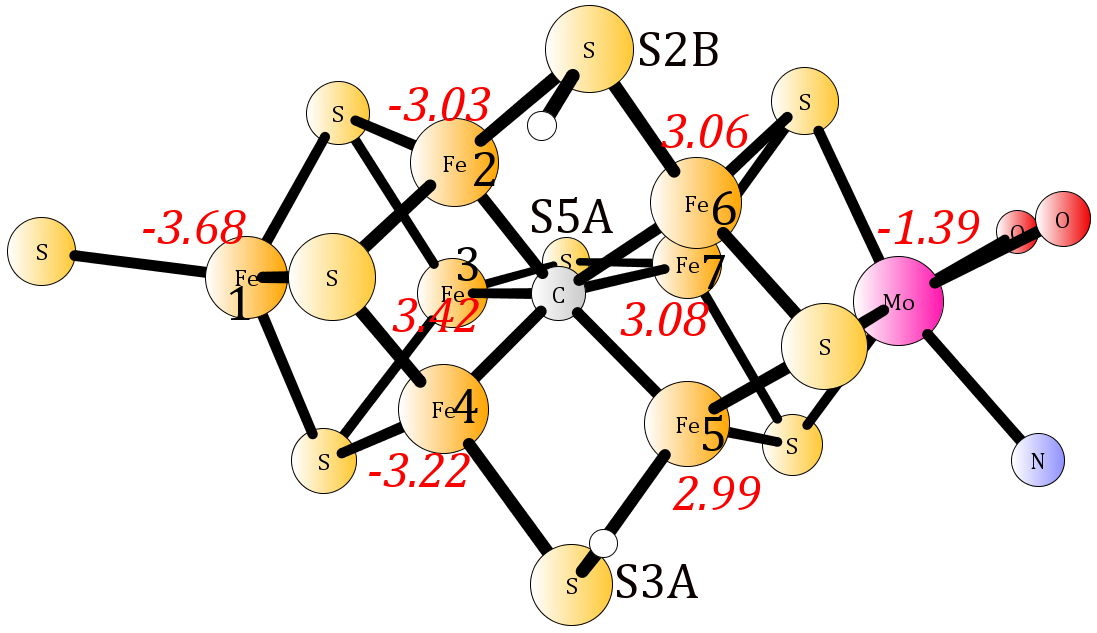


***Figure S4.*** *The TPSS optimized structure (C-H2),protons are on the S2B and S3A. Mulliken spin densities are shown in red italic. For clarity, only the core of the model is shown*


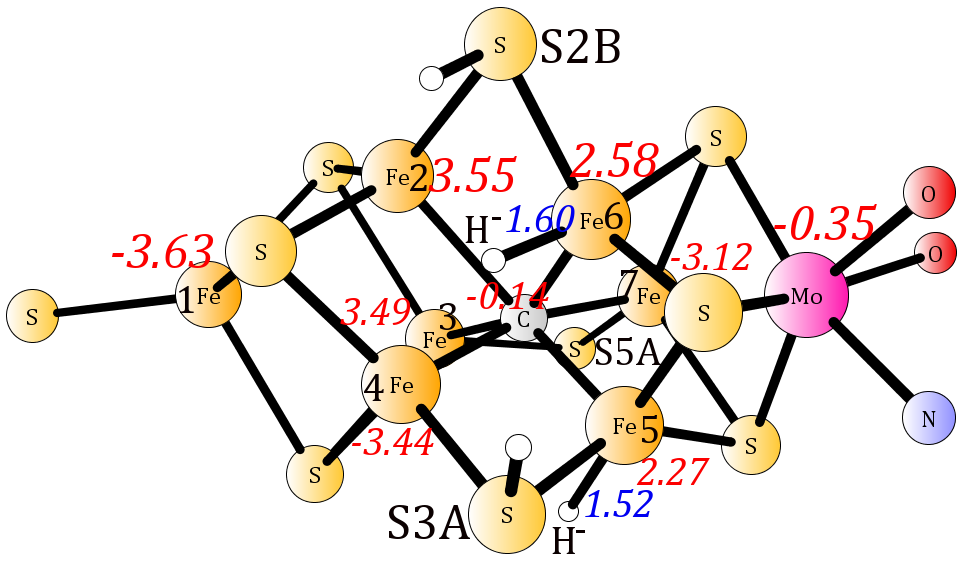


***Figure S5****. The TPSSh optimized structure (C, 2H^-^) with two hydrides and a central carbide.Distances (Å) between the iron and the hydrides are shown in blue italic. Mulliken spin densities are shown in red italic.* *For clarity, only the core of the model is shown.*


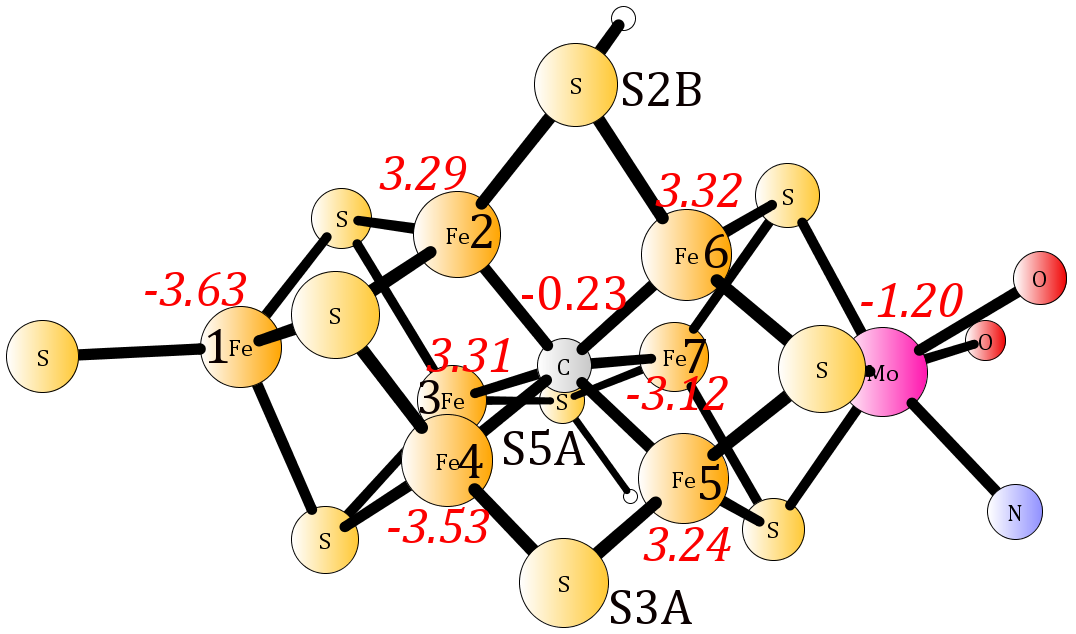


***Figure S6.*** *The TPSSh optimized structure (C-H2), protons are on the S2B and S5A. Mulliken spin densities are shown in red italic. For clarity, only the core of the model is shown.*


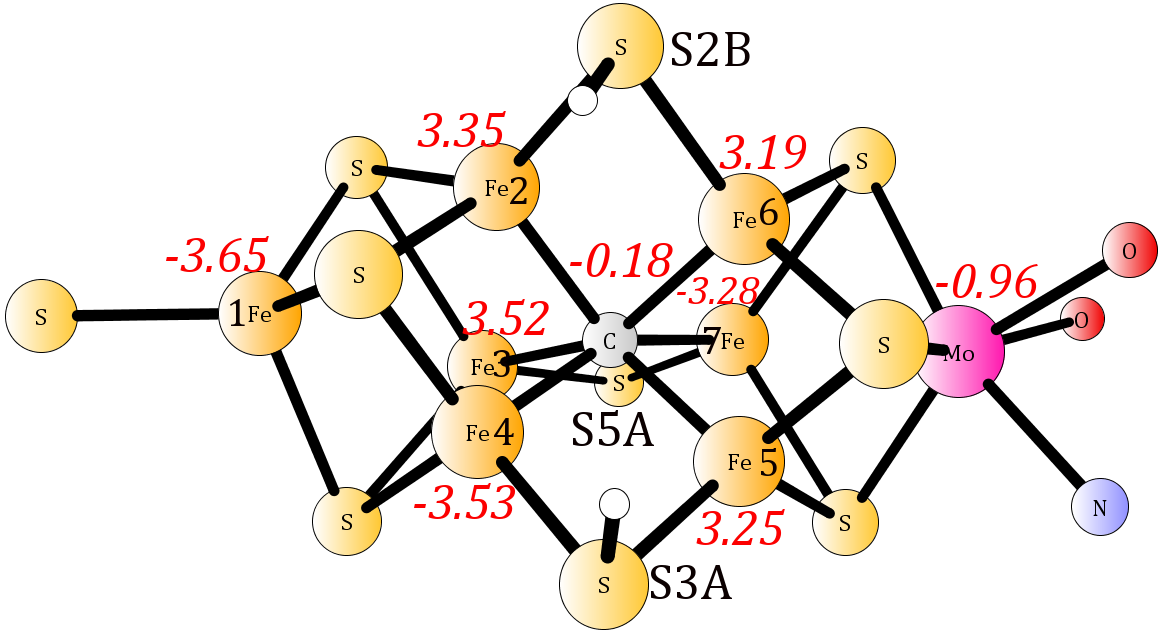


***Figure S7.*** *The TPSSh optimized structure (C-H2), protons are on the S2B and S3A Mulliken spin densities are shown in red italic. For clarity, only the core of the model is shown.*

**Coordinates for all structures**

**The TPSS optimized structure (C, 2H-) with two hydrides and a central carbide**

Mo1 16.02225 -6.09891 53.48593

Fe2 9.30762 -6.77961 55.62445

Fe3 11.76226 -5.63762 55.90271

Fe4 11.0521 -6.47694 53.52332

Fe5 11.72162 -8.24402 55.41731

Fe6 14.25912 -8.00133 54.47928

Fe7 14.15898 -5.64525 55.34005

Fe8 13.40823 -6.11689 52.88503

C9 17.60404 -2.446 54.32979

H10 17.82265 -1.48684 53.85184

H11 16.52861 -2.50505 54.54858

C12 17.98593 -3.60949 53.36963

C13 19.49008 -3.49777 52.98835

H14 20.08322 -3.73809 53.88152

H15 19.68457 -2.44656 52.73302

C16 19.90583 -4.40422 51.82285

H17 19.58357 -3.97487 50.86303

H18 19.38767 -5.36479 51.90687

C19 17.14259 -3.70164 52.06645

O20 16.7407 -4.92353 51.76403

O21 16.97956 -2.69911 51.36142

O22 17.75107 -4.85901 54.09055

C23 12.82109 -6.72466 54.59753

S24 10.86876 -7.1716 57.33512

S25 15.90752 -6.94077 55.69906

S26 9.88853 -4.76438 54.6544

S27 13.29836 -3.91061 56.63428

S28 13.32142 -9.8461 55.31574

S29 14.49642 -4.29342 53.55595

S30 9.82788 -8.39846 53.98177

S31 14.8589 -7.74825 52.28016

S32 11.89152 -6.70776 51.17057

H33 13.63502 -2.19016 49.04239

C34 13.29793 -2.02428 50.05533

H35 14.13945 -2.20142 50.73801

H36 12.88399 -1.0191 50.18428

N37 12.22057 -3.03449 50.188

H38 12.29092 -3.82541 49.54946

C39 11.51533 -3.27592 51.31569

N40 11.87586 -2.70412 52.47617

H41 11.34513 -3.01624 53.2987

H42 12.88403 -2.67288 52.65803

N43 10.36062 -3.97383 51.26232

H44 10.13925 -4.45514 50.39484

H45 10.0963 -4.4829 52.12632

H46 7.93595 0.29434 59.39576

C47 8.32484 -1.34072 60.87936

H48 8.29985 -1.37855 61.97892

H49 7.45147 -1.91368 60.52998

C50 9.58236 -2.05193 60.4494

N51 10.59039 -2.42176 61.34798

C52 9.90274 -2.51528 59.18751

H53 9.38327 -2.47516 58.2392

C54 11.48617 -3.09063 60.61847

H55 12.39848 -3.53942 60.99115

N56 11.11075 -3.16645 59.31296

H57 18.76632 -4.39586 57.59358

H58 16.69413 -8.91491 50.73656

C59 18.28424 -7.42179 50.73925

H60 17.60655 -6.56231 50.70982

H61 19.16523 -7.16625 50.13268

C62 18.74509 -7.62752 52.15073

N63 17.93814 -7.41517 53.27147

C64 19.99414 -8.02766 52.57654

H65 20.90323 -8.21974 52.0254

C66 18.69367 -7.67852 54.33898

H67 18.38317 -7.58307 55.36914

N68 19.94158 -8.05857 53.9605

H69 20.72389 -8.20672 54.58988

H70 6.39298 -9.48819 56.52584

C71 7.30628 -8.95899 56.8349

H72 7.42341 -9.03779 57.92319

H73 8.17041 -9.42554 56.34746

S74 7.18294 -7.17833 56.33798

H75 11.6085 -3.62929 58.54836

H76 17.79544 -4.61782 55.08917

H77 8.69335 -10.93649 62.45755

C78 18.38373 -2.58402 55.65525

O79 18.16113 -3.69539 56.29151

O80 19.19086 -1.69381 56.04404

C81 21.3939 -4.74944 51.72024

O82 21.70642 -5.65171 50.88881

O83 22.23709 -4.14436 52.49106

H84 15.70248 -0.19968 61.49125

C85 14.85492 -0.0829 60.83198

H86 13.91654 -0.28677 61.3261

H87 14.83229 0.95963 60.47648

C88 15.01058 -1.06216 59.64752

H89 14.19518 -0.90334 58.92624

H90 14.90689 -2.08949 60.02321

C91 16.34954 -0.96695 58.89427

H92 16.57756 0.07594 58.61917

H93 16.27908 -1.52365 57.94666

C94 17.53581 -1.53991 59.68653

O95 17.42603 -1.98272 60.8407

N96 18.74689 -1.49385 59.03976

H97 19.48212 -2.0391 59.48313

H98 18.81487 -1.43209 58.01558

C99 10.65188 0.00996 53.34607

H100 11.16378 0.95929 53.28716

H101 9.58636 0.185 53.35797

H102 10.9363 -0.61397 52.49662

C103 11.06566 -0.59194 54.71478

H104 10.77457 -1.65267 54.7625

C105 10.31598 0.1267 55.85539

H106 9.22725 0.00253 55.7544

H107 10.53487 1.20693 55.84849

H108 10.61389 -0.26999 56.83624

C109 12.58634 -0.47421 54.93442

H110 13.16029 -0.91923 54.10991

H111 12.89888 -0.97496 55.85949

H112 12.87694 0.58736 54.99797

H113 13.45503 -12.23066 53.18822

C114 13.91124 -11.62076 52.42219

H115 14.82406 -11.14898 52.79362

H116 14.10059 -12.19668 51.51177

N117 12.90207 -10.57264 52.17891

H118 12.70803 -10.04786 53.05867

C119 11.92435 -10.64214 51.27182

N120 12.19111 -11.19001 50.03397

H121 11.42977 -11.13948 49.36245

H122 13.10378 -10.96167 49.64614

N123 10.68192 -10.24972 51.57074

H124 10.06595 -9.72585 50.91741

H125 10.52238 -9.90992 52.53869

H126 19.18206 -8.21386 61.71535

C127 19.59783 -7.22995 61.99

H128 20.64143 -7.38009 62.29263

C129 18.83466 -6.66567 63.18747

O130 19.38338 -6.16063 64.17405

C131 19.49201 -6.27171 60.79912

H132 19.99995 -5.32959 61.05169

H133 18.44124 -6.00565 60.6341

C134 20.04596 -6.81831 59.47216

H135 19.52048 -7.75065 59.20605

H136 21.11207 -7.07565 59.53796

C137 19.8834 -5.87617 58.27519

O138 20.56472 -6.01304 57.24828

O139 18.95399 -4.94234 58.45211

N140 17.47403 -6.74708 63.02817

H141 17.11289 -7.08666 62.13861

C142 16.53683 -6.00589 63.85308

H143 15.82357 -6.70585 64.31627

H144 17.13038 -5.54441 64.65059

C145 15.75639 -4.93609 63.04268

H146 16.44067 -4.13531 62.73177

H147 15.00424 -4.49257 63.71494

C148 15.08814 -5.54531 61.8219

C149 13.98469 -6.41288 61.95759

H150 13.55982 -6.58488 62.94861

C151 15.62066 -5.33192 60.53515

H152 16.45393 -4.64002 60.41123

C153 13.43338 -7.05367 60.83978

H154 12.57476 -7.71515 60.95424

C155 15.07757 -5.98435 59.41563

H156 15.50239 -5.82168 58.42524

C157 13.98397 -6.85041 59.56448

H158 13.55491 -7.34598 58.6952

H159 9.00372 0.70505 60.77549

C160 8.127 0.15607 60.46472

H161 7.2837 0.45926 61.06784

O162 11.42329 -3.23122 64.08657

H163 12.22163 -2.78036 64.43242

H164 10.78522 -6.15873 62.84537

C165 10.41123 -7.00617 63.4012

H166 10.10401 -7.79158 62.70227

H167 11.27751 -7.4189 63.94861

N168 9.36228 -6.63286 64.35329

H169 9.34636 -5.64004 64.66967

C170 9.04435 -7.50038 65.34974

N171 9.23294 -8.81629 65.2249

H172 9.2862 -9.4045 66.1017

H173 9.49383 -9.23456 64.33496

N174 8.47148 -7.03558 66.48849

H175 8.75913 -6.10182 66.78243

H176 8.19918 -7.75094 67.2009

O177 6.77072 -3.52028 65.66767

H178 6.48191 -3.28046 64.76575

H179 6.60587 -4.48433 65.7178

O180 9.47772 -4.21834 65.73982

H181 8.63731 -3.70427 65.65762

H182 10.17763 -3.73628 65.2115

C183 8.62201 -12.88899 68.81298

H184 9.44243 -12.54996 69.46612

H185 8.05012 -13.63938 69.37178

C186 9.25201 -13.65328 67.62562

O187 9.25552 -14.89417 67.57775

C188 7.68969 -11.67739 68.49683

H189 7.08589 -11.45573 69.38784

H190 6.96887 -11.97561 67.71804

C191 8.28744 -10.31856 68.02327

O192 9.45547 -10.315 67.46738

O193 7.56397 -9.29458 68.15279

N194 9.82376 -12.85414 66.67829

H195 9.84659 -11.83634 66.9034

C196 10.618 -13.38698 65.58302

H197 10.12528 -14.28783 65.19775

H198 11.61984 -13.68782 65.93614

C199 10.78838 -12.35397 64.46064

H200 11.58808 -12.68881 63.78068

H201 11.10568 -11.37899 64.85307

C202 9.5471 -12.18958 63.60166

O203 8.71261 -13.03937 63.35503

O204 9.48439 -10.92587 63.04469

H205 11.14061 -2.76852 63.24471

H206 13.59178 -4.44758 57.84589

C207 27.69197 -4.71608 52.70401

H208 28.33163 -4.08075 52.07681

C209 27.29988 -3.95739 53.99459

H210 26.70931 -4.63284 54.6314

H211 28.22938 -3.73156 54.53355

C212 26.49672 -2.67725 53.69752

H213 27.10227 -2.01711 53.05709

H214 25.60667 -2.93197 53.10568

C215 26.06964 -1.86252 54.93898

H216 25.55821 -0.95047 54.59575

H217 26.96346 -1.53213 55.49198

C218 25.15791 -2.57025 55.95741

H219 25.66305 -3.42568 56.42229

H220 24.87679 -1.87504 56.75395

N221 23.8884 -3.08228 55.35377

H222 23.12625 -3.27876 56.05129

H223 23.40852 -2.40079 54.68362

H224 24.04454 -3.97298 54.80831

C225 24.52295 -7.55905 51.27299

H226 23.61749 -7.95589 51.76588

O227 25.61917 -8.15807 51.33389

N228 24.30513 -6.3967 50.63803

H229 23.32791 -5.99386 50.68919

C230 25.36514 -5.75622 49.8659

H231 24.95256 -4.82338 49.46108

H232 25.66924 -6.39072 49.02337

C233 26.64588 -5.4259 50.62908

O234 27.73382 -5.3957 50.03468

N235 26.49713 -5.04244 51.92885

H236 25.61369 -5.20688 52.44146

O237 22.26827 -1.69528 53.64046

H238 21.47673 -1.66797 54.21818

H239 22.1327 -2.5482 53.11901

O240 21.35259 -3.5397 56.17713

H241 20.65021 -2.86779 56.36669

H242 20.97305 -4.41433 56.44202

O243 24.23442 -5.30394 53.70078

H244 24.03976 -6.22075 54.00438

H245 23.44392 -4.98803 53.12499

O246 23.11471 -2.79722 58.4415

C247 23.57795 -3.15407 59.50984

H248 24.50355 -3.70672 59.57571

H249 23.11416 -2.87677 60.44516

C250 28.51 -5.95808 53.05399

O251 29.53716 -5.83246 53.74605

N252 28.01895 -7.17119 52.67085

H253 27.1952 -7.24625 52.06433

C254 28.53502 -8.42394 53.207

H255 29.24098 -8.15745 54.00354

H256 29.0857 -8.98412 52.43549

C257 27.35562 -9.29067 53.71447

H258 27.73421 -10.23444 54.13016

H259 26.72276 -9.53203 52.85227

C260 26.4976 -8.53938 54.77464

H261 26.53986 -7.46159 54.59166

H262 26.88609 -8.73386 55.78501

C263 25.03515 -8.92712 54.74729

O264 24.85196 -10.25842 54.97197

O265 24.07615 -8.17922 54.55859

H266 23.88077 -10.40748 54.91264

H267 12.26248 -7.99827 50.98773

H268 13.73519 -6.32385 56.64041

H269 18.25929 -9.49875 50.15036

C270 17.58407 -8.65608 50.15222

H271 17.27729 -8.44194 49.13875

H272 12.19983 -5.11659 53.00522

O273 14.79692 -5.54206 49.33906

O274 8.90896 -8.26076 50.80696

H275 15.44296 -5.20481 50.00669

H276 14.19275 -6.06559 49.90889

H277 8.65293 -8.31457 51.76119

H278 9.63937 -7.59477 50.86237

**The TPSS optimized structure (C-H2), protons are on the S2B and S5A**

Mo1 15.97827 -6.11559 53.46841

Fe2 9.21559 -7.53357 54.85956

Fe3 11.24061 -5.91095 55.32387

Fe4 11.16204 -7.2094 53.05599

Fe5 11.76666 -8.59286 55.24497

Fe6 14.21848 -8.04729 54.39049

Fe7 13.8912 -5.69639 55.21632

Fe8 13.44335 -6.13969 52.74339

C9 17.5589 -2.45992 54.3051

H10 17.81923 -1.51205 53.82563

H11 16.47157 -2.49069 54.46585

C12 17.95553 -3.64558 53.37903

C13 19.47408 -3.57005 53.04281

H14 20.02798 -3.83462 53.95319

H15 19.71068 -2.52341 52.80813

C16 19.89916 -4.48055 51.88363

H17 19.58993 -4.04837 50.9209

H18 19.37107 -5.43586 51.96314

C19 17.14844 -3.74121 52.05353

O20 16.71249 -4.9586 51.76516

O21 17.04389 -2.75661 51.31577

O22 17.67653 -4.88082 54.10984

C23 12.53909 -6.98795 54.46938

S24 10.44868 -7.54163 56.87735

S25 15.76834 -6.92236 55.72962

S26 9.63353 -5.5407 53.62408

S27 12.59524 -4.49476 56.80595

S28 13.54802 -9.95512 55.33679

S29 14.46891 -4.19435 53.55101

S30 10.24317 -9.26124 53.54142

S31 14.90296 -7.81394 52.16933

S32 11.83021 -6.32428 50.87793

H33 13.63515 -2.19026 49.04252

C34 13.29792 -2.02407 50.0551

H35 13.91494 -2.63541 50.72088

H36 13.38242 -0.96479 50.33022

N37 11.88934 -2.4717 50.08943

H38 11.34415 -2.31384 49.24843

C39 11.22832 -3.08066 51.09366

N40 11.73714 -3.20674 52.31807

H41 11.28858 -3.89999 52.93924

H42 12.7425 -3.09205 52.49326

N43 9.9349 -3.48336 50.87839

H44 9.76144 -3.88326 49.95793

H45 9.56909 -4.09303 51.6288

H46 7.93709 0.29786 59.39605

C47 8.35762 -1.33862 60.86805

H48 8.2703 -1.40252 61.9629

H49 7.54056 -1.95014 60.45417

C50 9.68363 -1.95283 60.49937

N51 10.64999 -2.30559 61.44744

C52 10.13492 -2.29917 59.24048

H53 9.68304 -2.23218 58.25992

C54 11.64938 -2.85024 60.74883

H55 12.55986 -3.26385 61.1644

N56 11.38286 -2.85618 59.41658

H57 18.68491 -4.42478 57.59137

H58 16.69615 -8.92131 50.73534

C59 18.2741 -7.43369 50.77361

H60 17.60345 -6.56968 50.74044

H61 19.17649 -7.1749 50.20099

C62 18.68433 -7.67725 52.19651

N63 17.85474 -7.45054 53.29862

C64 19.89692 -8.14902 52.65271

H65 20.81162 -8.38259 52.12706

C66 18.55691 -7.777 54.38337

H67 18.20178 -7.69942 55.40158

N68 19.79604 -8.21051 54.03324

H69 20.54479 -8.43334 54.68011

H70 6.39282 -9.48838 56.52585

C71 6.71236 -9.47832 55.47796

H72 7.64321 -10.0489 55.37818

H73 5.93633 -9.9528 54.8616

S74 6.94696 -7.73252 54.91083

H75 11.95427 -3.25578 58.6593

H76 17.73483 -4.62637 55.10966

H77 8.72347 -10.93214 62.43911

C78 18.25577 -2.56658 55.67939

O79 18.10826 -3.7106 56.27959

O80 18.92128 -1.60209 56.14775

C81 21.38478 -4.84411 51.78534

O82 21.68946 -5.728 50.93112

O83 22.23124 -4.27013 52.57514

H84 15.7025 -0.19965 61.49122

C85 14.85495 -0.08297 60.83202

H86 13.9165 -0.28673 61.32609

H87 14.83324 0.95956 60.47657

C88 15.00181 -1.06114 59.64666

H89 14.19831 -0.87994 58.91701

H90 14.87122 -2.0876 60.01626

C91 16.35299 -0.99004 58.91838

H92 16.6111 0.04932 58.65816

H93 16.29366 -1.53497 57.96363

C94 17.50737 -1.59787 59.73174

O95 17.36121 -2.05658 60.87612

N96 18.72658 -1.56388 59.10458

H97 19.44785 -2.1277 59.54783

H98 18.78774 -1.48933 58.07759

C99 10.65203 0.01013 53.34619

H100 11.16367 0.95927 53.28718

H101 9.58632 0.18496 53.35787

H102 10.91617 -0.58672 52.46522

C103 11.08356 -0.67661 54.66264

H104 10.72737 -1.71811 54.65288

C105 10.43755 0.03642 55.86713

H106 9.33895 -0.00815 55.8156

H107 10.73018 1.09853 55.89472

H108 10.75293 -0.4194 56.8159

C109 12.61682 -0.68577 54.80837

H110 13.10737 -1.15733 53.94635

H111 12.9276 -1.23872 55.70497

H112 13.00084 0.34401 54.89093

H113 13.455 -12.23057 53.18821

C114 13.91109 -11.62085 52.42217

H115 14.73789 -11.04381 52.85188

H116 14.23492 -12.22677 51.56785

N117 12.81284 -10.73559 51.99415

H118 12.16536 -10.40423 52.73082

C119 12.64059 -10.2461 50.77359

N120 13.67881 -10.32283 49.87197

H121 13.49731 -9.89855 48.96514

H122 14.57416 -10.02095 50.26315

N123 11.43302 -9.79529 50.36419

H124 10.68012 -9.65316 51.05726

H125 11.41237 -9.07968 49.64247

H126 19.18494 -8.21585 61.7191

C127 19.59789 -7.22993 61.99006

H128 20.64184 -7.3754 62.29474

C129 18.8367 -6.65317 63.18214

O130 19.3887 -6.12073 64.15275

C131 19.49189 -6.27175 60.79905

H132 19.93716 -5.30776 61.08891

H133 18.4355 -6.05692 60.59616

C134 20.12773 -6.74281 59.47712

H135 19.65018 -7.68163 59.15039

H136 21.20116 -6.95075 59.57758

C137 19.96461 -5.73822 58.32807

O138 20.79733 -5.64573 57.41486

O139 18.85205 -5.01608 58.42092

N140 17.47757 -6.7495 63.03424

H141 17.11146 -7.1234 62.16067

C142 16.53687 -6.00588 63.85305

H143 15.87054 -6.7093 64.37759

H144 17.13346 -5.47695 64.60494

C145 15.68486 -5.01942 63.01123

H146 16.31597 -4.19157 62.66104

H147 14.91491 -4.59434 63.67588

C148 15.0372 -5.70526 61.81723

C149 14.05375 -6.70138 61.99114

H150 13.70906 -6.94499 62.99839

C151 15.4678 -5.41113 60.50825

H152 16.22162 -4.6379 60.35734

C153 13.52042 -7.38497 60.88936

H154 12.7619 -8.1535 61.03982

C155 14.93503 -6.09507 59.40346

H156 15.27463 -5.85512 58.39712

C157 13.96215 -7.08846 59.58968

H158 13.55328 -7.62306 58.7322

H159 9.00377 0.70508 60.77522

C160 8.12695 0.1561 60.46484

H161 7.28373 0.4592 61.06791

O162 11.4144 -3.22525 64.10233

H163 12.22176 -2.78024 64.43265

H164 10.78526 -6.15879 62.84543

C165 10.41106 -7.00605 63.40104

H166 10.1041 -7.7917 62.7023

H167 11.27779 -7.41724 63.94841

N168 9.36128 -6.63299 64.35266

H169 9.3485 -5.64149 64.67298

C170 9.04157 -7.50215 65.34767

N171 9.2253 -8.81849 65.21989

H172 9.28025 -9.40739 66.09653

H173 9.49067 -9.23461 64.33023

N174 8.47107 -7.03762 66.48767

H175 8.76184 -6.10469 66.78179

H176 8.1998 -7.7533 67.20022

O177 6.77121 -3.51295 65.66946

H178 6.48191 -3.28046 64.76577

H179 6.60561 -4.47639 65.72806

O180 9.47697 -4.22477 65.7533

H181 8.6404 -3.70521 65.66813

H182 10.1836 -3.74194 65.2339

C183 8.62203 -12.88898 68.81296

H184 9.44215 -12.55024 69.46671

H185 8.05007 -13.63945 69.37162

C186 9.25279 -13.65313 67.62596

O187 9.26179 -14.89413 67.58136

C188 7.68933 -11.67762 68.49661

H189 7.08565 -11.45601 69.38769

H190 6.96864 -11.97688 67.71805

C191 8.28527 -10.31836 68.02191

O192 9.45211 -10.31378 67.46343

O193 7.56075 -9.2951 68.15147

N194 9.82183 -12.85413 66.67695

H195 9.84337 -11.83615 66.90066

C196 10.61798 -13.38699 65.58304

H197 10.124 -14.28531 65.19345

H198 11.61755 -13.69211 65.93889

C199 10.79547 -12.35169 64.46402

H200 11.59827 -12.68597 63.78744

H201 11.11226 -11.37798 64.86023

C202 9.5592 -12.1832 63.59853

O203 8.72102 -13.02893 63.35079

O204 9.50852 -10.92209 63.0343

H205 11.11053 -2.74071 63.27758

H206 12.44603 -5.4948 57.7111

C207 27.69197 -4.71607 52.70399

H208 28.32998 -4.08594 52.06982

C209 27.31324 -3.94956 53.99131

H210 26.73223 -4.62086 54.64077

H211 28.24915 -3.71725 54.51649

C212 26.50351 -2.67492 53.69359

H213 27.08891 -2.02532 53.02451

H214 25.5956 -2.94011 53.13435

C215 26.1154 -1.84511 54.93625

H216 25.56994 -0.94912 54.60401

H217 27.02548 -1.4901 55.44559

C218 25.26599 -2.55949 56.00107

H219 25.81144 -3.39565 56.45535

H220 25.00021 -1.8588 56.79864

N221 23.98099 -3.10542 55.45829

H222 23.258 -3.21678 56.19993

H223 23.47939 -2.46537 54.74733

H224 24.11173 -4.02875 54.95921

C225 24.52297 -7.55906 51.27299

H226 23.61797 -7.95535 51.76727

O227 25.6189 -8.15979 51.32517

N228 24.30571 -6.39245 50.64464

H229 23.32409 -6.00163 50.69951

C230 25.36499 -5.75606 49.86601

H231 24.95425 -4.82348 49.45905

H232 25.66636 -6.3948 49.02581

C233 26.64597 -5.42605 50.62901

O234 27.73371 -5.39051 50.03575

N235 26.49545 -5.0436 51.93116

H236 25.61932 -5.22393 52.44832

O237 22.27221 -1.86851 53.79614

H238 21.61028 -2.09622 54.49325

H239 22.1834 -2.66973 53.19257

O240 21.27525 -3.14179 56.17707

H241 20.528 -2.53795 56.42379

H242 20.97167 -4.04368 56.45367

O243 24.27094 -5.34234 53.80437

H244 24.10474 -6.26496 54.10876

H245 23.46779 -5.05801 53.23044

O246 23.11614 -2.7951 58.4404

C247 23.578 -3.15401 59.50997

H248 24.50353 -3.70674 59.57565

H249 23.11414 -2.87679 60.4451

C250 28.51 -5.95805 53.05401

O251 29.53984 -5.83193 53.74192

N252 28.01614 -7.17112 52.67448

H253 27.19036 -7.24701 52.07069

C254 28.53502 -8.42395 53.207

H255 29.26489 -8.15824 53.98186

H256 29.05754 -8.99485 52.42404

C257 27.36192 -9.27341 53.754

H258 27.73644 -10.23025 54.1426

H259 26.68743 -9.48861 52.91587

C260 26.57007 -8.51617 54.85992

H261 26.59087 -7.44128 54.65679

H262 27.02686 -8.69542 55.84374

C263 25.11444 -8.92005 54.92209

O264 24.95862 -10.23332 55.25221

O265 24.13928 -8.2022 54.70435

H266 23.98828 -10.39773 55.23723

H267 12.58712 -7.32993 50.3779

H268 18.25923 -9.49883 50.15064

C269 17.58408 -8.65574 50.15179

H270 17.27729 -8.44206 49.13885

O271 14.79687 -5.54221 49.33912

O272 8.90908 -8.26066 50.80695

H273 15.46706 -5.29944 50.0234

H274 14.00563 -5.71805 49.88992

H275 8.58963 -8.4551 51.71698

H276 9.47634 -7.47346 50.97247

**The TPSS optimized structure (C-H2), protons are on the S2B and S3A**

Mo1 16.04873 -6.0235 53.48616

Fe2 9.29842 -7.52279 54.96042

Fe3 11.28994 -5.8083 55.3576

Fe4 11.16751 -7.04313 53.03369

Fe5 11.82231 -8.43197 55.27185

Fe6 14.28656 -7.90983 54.33448

Fe7 13.94397 -5.60741 55.163

Fe8 13.51861 -6.11146 52.63085

C9 17.62898 -2.36228 54.34457

H10 17.90653 -1.4185 53.86703

H11 16.53963 -2.3802 54.49418

C12 18.01959 -3.55568 53.42683

C13 19.53806 -3.50348 53.0883

H14 20.09134 -3.7783 53.99589

H15 19.78917 -2.46037 52.85282

C16 19.94269 -4.41812 51.92403

H17 19.62905 -3.98047 50.96518

H18 19.40407 -5.36814 52.00416

C19 17.2234 -3.65748 52.0953

O20 16.80913 -4.884 51.80847

O21 17.11611 -2.6788 51.35255

O22 17.72241 -4.78032 54.1668

C23 12.57803 -6.86722 54.43513

S24 10.6057 -7.4282 56.97454

S25 15.82365 -6.82942 55.74999

S26 9.60415 -5.48136 53.7908

S27 12.69597 -4.35527 56.76085

S28 13.69975 -9.97154 55.41697

S29 14.50138 -4.14954 53.48439

S30 10.35468 -9.17546 53.58635

S31 15.04802 -7.72631 52.16661

S32 11.89964 -6.28951 50.98252

H33 13.63516 -2.19027 49.04252

C34 13.29793 -2.02404 50.05509

H35 13.92373 -2.6244 50.7233

H36 13.36284 -0.96167 50.3238

N37 11.8971 -2.49638 50.08505

H38 11.38189 -2.43523 49.21325

C39 11.23706 -3.09668 51.0931

N40 11.71081 -3.12498 52.34109

H41 11.31407 -3.85501 52.95173

H42 12.71158 -2.98025 52.50402

N43 9.96394 -3.55359 50.86051

H44 9.87377 -4.05403 49.97649

H45 9.62025 -4.15049 51.62847

H46 7.93718 0.29803 59.39612

C47 8.35784 -1.33866 60.86753

H48 8.26664 -1.40462 61.96191

H49 7.5441 -1.95154 60.44929

C50 9.68731 -1.94659 60.50212

N51 10.65072 -2.30154 61.45235

C52 10.14769 -2.27764 59.24254

H53 9.70144 -2.20379 58.25991

C54 11.65807 -2.83201 60.75469

H55 12.5693 -3.24274 61.17133

N56 11.39846 -2.82743 59.42104

H57 18.70726 -4.3545 57.63468

H58 16.7012 -8.92247 50.7391

C59 18.26805 -7.42208 50.76336

H60 17.58669 -6.56572 50.73227

H61 19.15403 -7.15516 50.16908

C62 18.71813 -7.63006 52.18161

N63 17.93892 -7.33641 53.30567

C64 19.94514 -8.0886 52.61322

H65 20.83776 -8.35136 52.06429

C66 18.68541 -7.60645 54.37576

H67 18.37606 -7.46542 55.40167

N68 19.90492 -8.07214 53.99818

H69 20.6827 -8.25832 54.62262

H70 6.39284 -9.48837 56.52589

C71 6.7957 -9.50271 55.50756

H72 7.746 -10.05 55.50049

H73 6.08494 -10.01784 54.84734

S74 7.02425 -7.76481 54.91945

H75 11.98149 -3.20607 58.66378

H76 17.75059 -4.51566 55.16582

H77 8.69882 -10.93389 62.45615

C78 18.31259 -2.47885 55.72561

O79 18.11473 -3.60987 56.3384

O80 19.01611 -1.53926 56.18722

C81 21.42263 -4.79461 51.81069

O82 21.70945 -5.68517 50.95765

O83 22.28357 -4.22392 52.58785

H84 15.70249 -0.19966 61.49121

C85 14.85495 -0.08295 60.83203

H86 13.9165 -0.28674 61.32609

H87 14.8333 0.95912 60.47569

C88 15.00728 -1.06284 59.64866

H89 14.20796 -0.88425 58.91393

H90 14.87624 -2.08868 60.01975

C91 16.362 -0.99308 58.92644

H92 16.61401 0.04325 58.64938

H93 16.30984 -1.55602 57.98162

C94 17.51716 -1.58158 59.75227

O95 17.36392 -2.05494 60.88995

N96 18.74614 -1.51473 59.14686

H97 19.47036 -2.06871 59.5975

H98 18.82668 -1.42738 58.1226

C99 10.65206 0.01017 53.3462

H100 11.16366 0.95926 53.28719

H101 9.58632 0.18492 53.35786

H102 10.91696 -0.59079 52.46919

C103 11.08634 -0.66689 54.66684

H104 10.75315 -1.71574 54.6561

C105 10.41808 0.03452 55.86602

H106 9.32129 -0.04041 55.81157

H107 10.68128 1.10448 55.8888

H108 10.74336 -0.40714 56.81828

C109 12.61862 -0.64071 54.82326

H110 13.12657 -1.0969 53.96288

H111 12.93725 -1.18744 55.72102

H112 12.97703 0.39809 54.9098

H113 13.45501 -12.23044 53.18829

C114 13.91108 -11.62098 52.42201

H115 14.72704 -11.03021 52.85781

H116 14.27489 -12.24255 51.59528

N117 12.81738 -10.75792 51.93857

H118 12.08151 -10.48441 52.61052

C119 12.83642 -10.04042 50.81135

N120 13.97138 -10.02115 50.04362

H121 13.91199 -9.38912 49.24643

H122 14.83141 -9.86668 50.57329

N123 11.7318 -9.42254 50.34209

H124 10.86663 -9.48174 50.89699

H125 11.88461 -8.43172 50.07115

H126 19.1882 -8.217 61.71749

C127 19.59788 -7.22993 61.99005

H128 20.64128 -7.37393 62.29682

C129 18.83253 -6.66173 63.18318

O130 19.38131 -6.14911 64.16575

C131 19.49191 -6.27175 60.79905

H132 19.94963 -5.3123 61.0838

H133 18.43596 -6.04338 60.60827

C134 20.10484 -6.74598 59.46826

H135 19.61648 -7.68026 59.14439

H136 21.17738 -6.96623 59.55331

C137 19.93918 -5.74073 58.31873

O138 20.72525 -5.70851 57.36128

O139 18.88144 -4.94742 58.46178

N140 17.47284 -6.74516 63.02517

H141 17.10947 -7.09511 62.14075

C142 16.53687 -6.00588 63.85305

H143 15.8502 -6.70979 64.34976

H144 17.1361 -5.51192 64.62633

C145 15.71691 -4.97599 63.03346

H146 16.37497 -4.16613 62.6911

H147 14.96771 -4.53409 63.71034

C148 15.03386 -5.62046 61.83762

C149 13.98844 -6.55077 62.01242

H150 13.62896 -6.76952 63.01988

C151 15.48368 -5.35404 60.52936

H152 16.2802 -4.6252 60.37815

C153 13.40781 -7.19371 60.91078

H154 12.59602 -7.90621 61.05854

C155 14.90608 -5.99968 59.42412

H156 15.25889 -5.77929 58.41768

C157 13.86667 -6.92289 59.61162

H158 13.40628 -7.41607 58.75502

H159 9.00377 0.70506 60.77523

C160 8.12692 0.15613 60.46486

H161 7.28371 0.45922 61.06789

O162 11.41697 -3.2293 64.10143

H163 12.22174 -2.78024 64.43267

H164 10.78527 -6.1588 62.84545

C165 10.41105 -7.00602 63.40101

H166 10.1041 -7.79171 62.70231

H167 11.27732 -7.41823 63.94867

N168 9.36134 -6.6337 64.35277

H169 9.34797 -5.64164 64.67227

C170 9.04538 -7.50246 65.34985

N171 9.23117 -8.81865 65.22251

H172 9.28486 -9.4084 66.09883

H173 9.4865 -9.23637 64.33078

N174 8.47815 -7.0382 66.49084

H175 8.76578 -6.10363 66.78286

H176 8.20608 -7.75371 67.20355

O177 6.77385 -3.50912 65.66957

H178 6.48192 -3.28046 64.76577

H179 6.60109 -4.47075 65.73535

O180 9.47709 -4.22644 65.75025

H181 8.64069 -3.70633 65.66557

H182 10.18471 -3.74319 65.23238

C183 8.62202 -12.88899 68.81297

H184 9.44316 -12.55277 69.4668

H185 8.04893 -13.6389 69.37117

C186 9.25079 -13.6531 67.62474

O187 9.25866 -14.89391 67.57903

C188 7.69144 -11.67568 68.49793

H189 7.08889 -11.45338 69.38955

H190 6.9695 -11.97356 67.72001

C191 8.28886 -10.31735 68.0231

O192 9.45639 -10.31373 67.46563

O193 7.5648 -9.29354 68.15051

N194 9.8227 -12.85384 66.67728

H195 9.84709 -11.83613 66.90295

C196 10.61799 -13.38699 65.58303

H197 10.12523 -14.28735 65.19669

H198 11.6193 -13.68866 65.93704

C199 10.79053 -12.35339 64.46154

H200 11.59015 -12.68899 63.7818

H201 11.10902 -11.37919 64.85518

C202 9.54972 -12.18666 63.60219

O203 8.71356 -13.03486 63.3561

O204 9.49028 -10.92385 63.0428

H205 11.10976 -2.74348 63.27839

H206 12.57731 -5.3448 57.68263

C207 27.69199 -4.71607 52.704

H208 28.33001 -4.08601 52.06969

C209 27.31802 -3.95147 53.99335

H210 26.73916 -4.62394 54.64337

H211 28.25641 -3.72185 54.51528

C212 26.50892 -2.675 53.7039

H213 27.09026 -2.02417 53.03256

H214 25.59606 -2.93657 53.15098

C215 26.13324 -1.84993 54.9535

H216 25.58148 -0.95415 54.63151

H217 27.04828 -1.49531 55.45403

C218 25.29747 -2.57242 56.02368

H219 25.84986 -3.41 56.46677

H220 25.03776 -1.87694 56.82783

N221 24.00865 -3.117 55.48878

H222 23.29006 -3.23496 56.23379

H223 23.50673 -2.46871 54.78676

H224 24.13625 -4.03628 54.98217

C225 24.52298 -7.55906 51.27299

H226 23.6186 -7.95264 51.77075

O227 25.6172 -8.16318 51.32257

N228 24.30679 -6.39192 50.64583

H229 23.32954 -5.99083 50.71155

C230 25.36501 -5.75607 49.866

H231 24.95399 -4.8236 49.45913

H232 25.6656 -6.39531 49.02601

C233 26.64597 -5.42605 50.62901

O234 27.73413 -5.39174 50.0366

N235 26.49535 -5.0423 51.93114

H236 25.62138 -5.22582 52.45034

O237 22.32436 -1.83466 53.82958

H238 21.64209 -2.04541 54.51011

H239 22.23171 -2.63381 53.22261

O240 21.30846 -3.18056 56.19888

H241 20.58464 -2.55642 56.46242

H242 20.97424 -4.07762 56.45271

O243 24.28964 -5.34671 53.82103

H244 24.10781 -6.27204 54.10815

H245 23.49591 -5.04121 53.24495

O246 23.11829 -2.79131 58.44153

C247 23.57802 -3.15397 59.50999

H248 24.50351 -3.70675 59.57565

H249 23.11413 -2.87681 60.44509

C250 28.50999 -5.95806 53.054

O251 29.53914 -5.83287 53.74296

N252 28.01643 -7.17129 52.67385

H253 27.19356 -7.24727 52.06635

C254 28.53502 -8.42395 53.207

H255 29.25067 -8.15812 53.99499

H256 29.07459 -8.98717 52.42993

C257 27.35918 -9.28569 53.7292

H258 27.73657 -10.23787 54.12626

H259 26.70535 -9.5096 52.87749

C260 26.53338 -8.53738 54.81605

H261 26.55624 -7.4609 54.6219

H262 26.96271 -8.7201 55.81192

C263 25.07708 -8.94418 54.84198

O264 24.91728 -10.27068 55.10832

O265 24.10347 -8.21508 54.65307

H266 23.94715 -10.43392 55.07644

H267 14.16283 -9.5303 56.61177

H268 18.25922 -9.49885 50.15064

C269 17.58414 -8.65571 50.15181

H270 17.27727 -8.44206 49.13885

O271 14.79692 -5.54221 49.33914

O272 8.90896 -8.26074 50.80692

H273 15.40525 -5.27944 50.06873

H274 14.00979 -5.88162 49.82521

H275 8.64049 -8.37591 51.74555

H276 9.54066 -7.50717 50.87508

**The TPSSh optimized structure (C, 2H-) with two hydrides and a central carbide.**

Mo1 15.83038 -5.86957 53.3868

Fe2 8.6263 -6.96491 54.65113

Fe3 11.04088 -5.58578 55.25194

Fe4 10.82582 -6.59523 52.74598

Fe5 11.39968 -8.1761 54.82627

Fe6 13.94097 -7.76393 54.17912

Fe7 13.57425 -5.22685 55.10059

Fe8 13.33522 -5.77092 52.47613

C9 17.64621 -2.38989 54.4024

H10 17.94822 -1.45361 53.93056

H11 16.56084 -2.37435 54.56419

C12 17.99659 -3.58873 53.48998

C13 19.5253 -3.63517 53.22053

H14 20.02455 -3.92904 54.14932

H15 19.84125 -2.61148 52.99054

C16 19.92545 -4.5706 52.07789

H17 19.61045 -4.15007 51.1147

H18 19.39017 -5.51644 52.17659

C19 17.27849 -3.62393 52.12273

O20 16.79469 -4.80818 51.81508

O21 17.28154 -2.64309 51.38321

O22 17.5582 -4.78773 54.18824

C23 12.38514 -6.56842 54.05134

S24 10.11933 -7.13225 56.57849

S25 15.3729 -6.65679 55.5827

S26 9.38763 -4.87525 53.68677

S27 14.25228 -3.81489 56.82676

S28 13.12981 -9.66061 55.06419

S29 14.41569 -3.95909 53.37608

S30 9.76783 -8.61139 53.14271

S31 14.794 -7.49859 52.00697

S32 11.76747 -5.83659 50.62284

H33 13.6352 -2.19019 49.04254

C34 13.29791 -2.02413 50.05503

H35 13.80536 -2.74807 50.69746

H36 13.53961 -1.00622 50.3771

N37 11.849 -2.26317 50.06207

H38 11.33672 -2.05812 49.21556

C39 11.13103 -2.80657 51.0595

N40 11.62156 -2.96426 52.27741

H41 11.10393 -3.56805 52.93592

H42 12.61103 -2.83342 52.48414

N43 9.81776 -3.10755 50.83712

H44 9.63066 -3.53024 49.9336

H45 9.39436 -3.64771 51.60644

H46 7.93476 0.32819 59.4029

C47 8.35859 -1.35082 60.80775

H48 8.25548 -1.45802 61.89495

H49 7.54659 -1.93973 60.36118

C50 9.68109 -1.98901 60.44998

N51 10.63143 -2.31542 61.41797

C52 10.1214 -2.43417 59.22065

H53 9.67183 -2.41475 58.23946

C54 11.6071 -2.93612 60.76625

H55 12.502 -3.34989 61.20789

N56 11.3458 -3.02664 59.44003

H57 18.7368 -4.44593 57.60628

H58 16.6867 -8.95167 50.70762

C59 18.20806 -7.42757 50.82573

H60 17.53637 -6.56938 50.745

H61 19.14904 -7.15787 50.33132

C62 18.49673 -7.67511 52.27708

N63 17.62882 -7.34511 53.31542

C64 19.61924 -8.25603 52.8171

H65 20.53639 -8.58986 52.3588

C66 18.21724 -7.72135 54.44211

H67 17.80818 -7.5935 55.43225

N68 19.42173 -8.2819 54.18357

H69 20.08695 -8.58442 54.88101

H70 6.39285 -9.48834 56.52589

C71 6.59784 -9.35104 55.45894

H72 7.60029 -9.72728 55.23615

H73 5.87011 -9.92892 54.87933

S74 6.45227 -7.56766 55.00643

H75 11.94123 -3.47571 58.74547

H76 17.66188 -4.56394 55.18402

H77 8.77638 -10.90664 62.44575

C78 18.32246 -2.52981 55.77845

O79 18.2177 -3.6968 56.32196

O80 18.93449 -1.56293 56.29513

C81 21.40343 -4.93719 51.95482

O82 21.68923 -5.83836 51.1205

O83 22.26335 -4.33594 52.69677

H84 15.70247 -0.19963 61.49124

C85 14.85501 -0.083 60.83198

H86 13.91649 -0.28673 61.32611

H87 14.8371 0.95658 60.47681

C88 14.97582 -1.066 59.65433

H89 14.20157 -0.84147 58.90972

H90 14.77559 -2.07934 60.01851

C91 16.33675 -1.08618 58.95669

H92 16.66426 -0.07512 58.67642

H93 16.25683 -1.6561 58.02152

C94 17.42765 -1.74681 59.80137

O95 17.21188 -2.25919 60.9049

N96 18.67713 -1.70426 59.24647

H97 19.35452 -2.3165 59.68657

H98 18.78393 -1.57566 58.23291

C99 10.65199 0.01001 53.34608

H100 11.16373 0.95928 53.28717

H101 9.58633 0.18501 53.35795

H102 10.91645 -0.57962 52.46754

C103 11.10929 -0.6514 54.66019

H104 10.82235 -1.71099 54.65476

C105 10.4192 0.01849 55.85912

H106 9.32936 -0.10092 55.80671

H107 10.63947 1.0952 55.88488

H108 10.7625 -0.41195 56.80737

C109 12.63495 -0.54727 54.79836

H110 13.15699 -1.00538 53.95096

H111 12.99657 -1.04424 55.70376

H112 12.93998 0.50809 54.84676

H113 13.45498 -12.23048 53.18831

C114 13.9111 -11.62094 52.42198

H115 14.79243 -11.11621 52.82845

H116 14.1667 -12.22835 51.54956

N117 12.86891 -10.66191 52.06114

H118 12.40839 -10.22614 52.88199

C119 12.69905 -10.1101 50.87295

N120 13.6403 -10.30709 49.89371

H121 13.45549 -9.83135 49.01807

H122 14.59844 -10.18003 50.20851

N123 11.55228 -9.47364 50.57883

H124 10.88579 -9.30555 51.35934

H125 11.59528 -8.6949 49.93102

H126 19.20742 -8.22011 61.71295

C127 19.59792 -7.22988 61.99005

H128 20.63755 -7.35572 62.30796

C129 18.81994 -6.67171 63.17532

O130 19.36373 -6.1932 64.17196

C131 19.49183 -6.27179 60.79904

H132 19.86819 -5.28944 61.1147

H133 18.43984 -6.11089 60.54534

C134 20.22175 -6.69637 59.51711

H135 19.81569 -7.65296 59.15645

H136 21.295 -6.84552 59.67695

C137 20.06374 -5.68182 58.38338

O138 20.9531 -5.48361 57.55222

O139 18.88989 -5.07684 58.40304

N140 17.46923 -6.71228 62.99759

H141 17.0974 -7.07476 62.12633

C142 16.53691 -6.00587 63.85302

H143 15.98498 -6.72258 64.47818

H144 17.13557 -5.37956 64.5197

C145 15.54908 -5.15791 63.02736

H146 16.09019 -4.33774 62.54218

H147 14.83328 -4.70705 63.73072

C148 14.80331 -5.96567 61.97646

C149 14.02046 -7.07834 62.33455

H150 13.9373 -7.36304 63.38269

C151 14.89465 -5.62677 60.61614

H152 15.50042 -4.77331 60.31778

C153 13.34445 -7.82209 61.36292

H154 12.74792 -8.6823 61.65983

C155 14.20903 -6.36387 59.64088

H156 14.27351 -6.06957 58.59793

C157 13.43104 -7.46396 60.01228

H158 12.89775 -8.02834 59.25229

H159 9.00369 0.70515 60.77541

C160 8.12715 0.15595 60.46485

H161 7.28364 0.45923 61.06779

O162 11.37667 -3.16624 64.13957

H163 12.22172 -2.78015 64.43267

H164 10.78539 -6.15879 62.84559

C165 10.41083 -7.0062 63.4011

H166 10.10413 -7.79167 62.70219

H167 11.28077 -7.4129 63.93921

N168 9.37058 -6.64035 64.35848

H169 9.33876 -5.6521 64.66483

C170 9.06449 -7.50427 65.35469

N171 9.26413 -8.81399 65.23016

H172 9.30793 -9.402 66.10015

H173 9.53177 -9.22592 64.3438

N174 8.49942 -7.04453 66.49135

H175 8.75907 -6.10268 66.77254

H176 8.22555 -7.75251 67.20147

O177 6.74621 -3.50338 65.67425

H178 6.4819 -3.28047 64.76577

H179 6.55536 -4.45577 65.74978

O180 9.44286 -4.23094 65.7666

H181 8.61247 -3.70995 65.6965

H182 10.14764 -3.7361 65.2717

C183 8.6222 -12.88888 68.81268

H184 9.43788 -12.5564 69.47014

H185 8.04706 -13.63968 69.36232

C186 9.25869 -13.64891 67.62859

O187 9.27207 -14.88409 67.58874

C188 7.69858 -11.67863 68.49337

H189 7.09455 -11.45423 69.37999

H190 6.98054 -11.97285 67.71465

C191 8.29952 -10.32535 68.02227

O192 9.46203 -10.3243 67.47189

O193 7.58035 -9.3058 68.14996

N194 9.82539 -12.85684 66.6784

H195 9.84735 -11.84375 66.8987

C196 10.61785 -13.387 65.58324

H197 10.11648 -14.27176 65.18055

H198 11.60911 -13.70722 65.9389

C199 10.80832 -12.34172 64.48251

H200 11.61479 -12.66653 63.81035

H201 11.12179 -11.37604 64.89312

C202 9.58419 -12.15986 63.61019

O203 8.73868 -12.99143 63.36643

O204 9.55481 -10.91094 63.04114

H205 11.09837 -2.70432 63.30387

H206 13.24343 -2.92201 56.72411

C207 27.69196 -4.71606 52.70398

H208 28.33034 -4.09043 52.07073

C209 27.3179 -3.95136 53.98586

H210 26.74708 -4.62218 54.63982

H211 28.25388 -3.71103 54.50204

C212 26.50086 -2.68676 53.69076

H213 27.06448 -2.04718 52.99827

H214 25.58009 -2.96052 53.16277

C215 26.14798 -1.84566 54.92982

H216 25.58206 -0.96255 54.60665

H217 27.06839 -1.47616 55.40305

C218 25.34459 -2.55803 56.02408

H219 25.91513 -3.37832 56.4704

H220 25.09202 -1.85317 56.81947

N221 24.05752 -3.12442 55.52469

H222 23.36202 -3.21643 56.28691

H223 23.54455 -2.50319 54.81179

H224 24.18193 -4.0523 55.04436

C225 24.52299 -7.55905 51.273

H226 23.61957 -7.94202 51.77274

O227 25.6097 -8.16392 51.31643

N228 24.31266 -6.39152 50.64884

H229 23.34221 -6.00309 50.73777

C230 25.36496 -5.75604 49.866

H231 24.95743 -4.82432 49.46196

H232 25.6654 -6.39365 49.02864

C233 26.64602 -5.42607 50.629

O234 27.72649 -5.38874 50.03586

N235 26.50059 -5.0481 51.92965

H236 25.63204 -5.23554 52.44641

O237 22.35375 -1.90971 53.85687

H238 21.70807 -2.12455 54.57039

H239 22.24786 -2.70922 53.26649

O240 21.32254 -3.00472 56.2255

H241 20.57473 -2.41449 56.48441

H242 21.04859 -3.90102 56.52865

O243 24.33696 -5.37415 53.878

H244 24.18924 -6.29221 54.18869

H245 23.51953 -5.10206 53.33132

O246 23.13033 -2.78359 58.44488

C247 23.57801 -3.15398 59.50998

H248 24.50352 -3.70676 59.57565

H249 23.11413 -2.8768 60.44509

C250 28.51001 -5.95807 53.05403

O251 29.54314 -5.82927 53.72504

N252 28.01001 -7.16914 52.69161

H253 27.1823 -7.24276 52.09789

C254 28.53501 -8.42394 53.20699

H255 29.2948 -8.16682 53.95129

H256 29.01849 -8.99888 52.40641

C257 27.37895 -9.25262 53.80105

H258 27.75043 -10.21981 54.15837

H259 26.65991 -9.44298 52.99716

C260 26.67113 -8.49179 54.95212

H261 26.6819 -7.42085 54.7414

H262 27.19025 -8.6708 55.90085

C263 25.22649 -8.89213 55.09919

O264 25.08698 -10.16984 55.5317

O265 24.24878 -8.19902 54.8537

H266 24.123 -10.33833 55.56199

H267 12.51586 -6.86142 50.15844

H268 12.49876 -5.64649 56.27498

H269 18.25936 -9.49869 50.15042

C270 17.58366 -8.65599 50.15231

H271 17.27753 -8.442 49.13867

H272 12.25082 -4.2626 54.99158

**The TPSSh optimized structure (C, 2H-) with two hydrides and a central carbide, the same structure in our previous stydy.**

Mo1 16.11255 -5.86198 53.47612

Fe2 8.96639 -7.23464 54.78645

Fe3 11.27544 -5.60893 55.33079

Fe4 11.11096 -6.79511 52.83168

Fe5 11.95973 -8.17984 55.14219

Fe6 14.43638 -7.95819 53.96751

Fe7 13.87452 -5.80655 55.17537

Fe8 13.55381 -5.7835 52.45799

C9 17.80435 -2.23703 54.54326

H10 18.10322 -1.29944 54.07283

H11 16.72547 -2.22098 54.73549

C12 18.11881 -3.41821 53.59364

C13 19.62614 -3.42482 53.2324

H14 20.19561 -3.71916 54.12107

H15 19.8953 -2.38922 52.99395

C16 19.98834 -4.32807 52.05094

H17 19.64733 -3.88143 51.10869

H18 19.46009 -5.28015 52.13102

C19 17.31898 -3.42775 52.27232

O20 16.89084 -4.6193 51.93203

O21 17.22782 -2.40835 51.59186

O22 17.7559 -4.6405 54.29506

C23 12.7731 -6.7741 54.00597

S24 10.45052 -7.23825 56.73888

S25 15.85124 -6.92735 55.55598

S26 9.59853 -5.11162 53.79972

S27 12.83037 -4.2321 56.71321

S28 13.91349 -9.73725 55.39076

S29 14.49782 -4.08168 53.71673

S30 10.34324 -8.9156 53.50309

S31 15.16636 -7.34732 51.94347

S32 11.9783 -6.03613 50.81753

H33 13.63516 -2.19022 49.04257

C34 13.29794 -2.0241 50.05501

H35 13.89305 -2.66561 50.7107

H36 13.43131 -0.97445 50.33675

N37 11.88572 -2.41724 50.09132

H38 11.36586 -2.35753 49.22689

C39 11.22401 -2.97032 51.11982

N40 11.72376 -2.99365 52.34961

H41 11.28909 -3.66154 53.00181

H42 12.71744 -2.84268 52.50978

N43 9.93718 -3.38024 50.91766

H44 9.82896 -3.91468 50.05984

H45 9.58055 -3.93166 51.71175

H46 7.93693 0.30403 59.39978

C47 8.36812 -1.3346 60.85327

H48 8.27918 -1.41343 61.94407

H49 7.56445 -1.95179 60.4297

C50 9.70374 -1.91705 60.48043

N51 10.66808 -2.27258 61.42163

C52 10.17146 -2.20989 59.21924

H53 9.72666 -2.12039 58.24033

C54 11.68271 -2.76519 60.71784

H55 12.59955 -3.16538 61.12605

N56 11.4281 -2.73686 59.38843

H57 18.85232 -4.27166 57.71204

H58 16.70985 -8.91654 50.74864

C59 18.26949 -7.40314 50.72582

H60 17.58624 -6.54952 50.6834

H61 19.13707 -7.14916 50.10405

C62 18.75254 -7.55073 52.13636

N63 18.02748 -7.14704 53.25512

C64 19.97783 -8.01078 52.55563

H65 20.83935 -8.34087 51.99846

C66 18.80563 -7.35322 54.31006

H67 18.55672 -7.12038 55.33147

N68 19.99116 -7.88148 53.93048

H69 20.77848 -8.04077 54.54537

H70 6.39284 -9.48834 56.5259

C71 6.76065 -9.50246 55.49603

H72 7.76758 -9.92928 55.47904

H73 6.10146 -10.13424 54.89099

S74 6.75725 -7.79037 54.81479

H75 12.02223 -3.0802 58.62895

H76 17.74682 -4.413 55.28367

H77 8.64027 -10.94647 62.50281

C78 18.55094 -2.44965 55.86818

O79 18.18302 -3.50697 56.51686

O80 19.46882 -1.67614 56.24151

C81 21.4663 -4.67673 51.89786

O82 21.74997 -5.57436 51.06119

O83 22.33051 -4.06985 52.6333

H84 15.7025 -0.19964 61.49123

C85 14.85493 -0.08302 60.83204

H86 13.91652 -0.2867 61.32606

H87 14.83259 0.95867 60.48275

C88 15.03029 -1.05022 59.64589

H89 14.25389 -0.86356 58.89351

H90 14.88929 -2.07724 60.00048

C91 16.40169 -0.97772 58.9599

H92 16.66316 0.05792 58.69949

H93 16.3716 -1.53394 58.01324

C94 17.53044 -1.57576 59.80477

O95 17.34129 -2.0688 60.92125

N96 18.78155 -1.50197 59.2533

H97 19.47435 -2.07964 59.71606

H98 18.92004 -1.40005 58.24536

C99 10.65202 0.01003 53.34599

H100 11.16368 0.95927 53.28728

H101 9.58632 0.185 53.35793

H102 10.91772 -0.58975 52.47367

C103 11.08524 -0.65313 54.66875

H104 10.76825 -1.70379 54.66589

C105 10.40037 0.04613 55.85403

H106 9.30865 -0.0548 55.79902

H107 10.63847 1.11951 55.86321

H108 10.73375 -0.37427 56.80975

C109 12.61096 -0.59885 54.83701

H110 13.13386 -1.03841 53.98064

H111 12.93342 -1.14449 55.73028

H112 12.94714 0.44408 54.93067

H113 13.45508 -12.23044 53.18828

C114 13.91098 -11.62097 52.42202

H115 14.67075 -10.97254 52.87424

H116 14.33631 -12.25186 51.63576

N117 12.78157 -10.84532 51.89198

H118 12.03747 -10.61848 52.56193

C119 12.87914 -9.95011 50.90077

N120 14.0278 -9.86759 50.17566

H121 14.08125 -9.0406 49.58782

H122 14.87816 -9.98161 50.71943

N123 11.8108 -9.22746 50.53449

H124 11.03463 -9.25773 51.21014

H125 11.99701 -8.23407 50.28388

H126 19.18576 -8.21236 61.71646

C127 19.59783 -7.22989 61.98999

H128 20.63803 -7.37551 62.29659

C129 18.83134 -6.66448 63.18207

O130 19.37991 -6.15867 64.16117

C131 19.49191 -6.27178 60.79918

H132 19.99049 -5.32901 61.05506

H133 18.4422 -6.01045 60.63346

C134 20.0523 -6.80871 59.4798

H135 19.56272 -7.75566 59.21222

H136 21.1245 -7.03297 59.54382

C137 19.85812 -5.87488 58.28769

O138 20.38876 -6.10924 57.19735

O139 19.08945 -4.83359 58.54694

N140 17.47503 -6.74359 63.02844

H141 17.1121 -7.09427 62.14838

C142 16.53691 -6.00589 63.85302

H143 15.84849 -6.70828 64.34269

H144 17.12961 -5.51496 64.62911

C145 15.73189 -4.97455 63.03006

H146 16.39804 -4.17232 62.694

H147 14.98495 -4.52536 63.70017

C148 15.04635 -5.60107 61.82891

C149 13.99603 -6.52144 61.99247

H150 13.64419 -6.75851 62.9956

C151 15.48514 -5.31233 60.52552

H152 16.28636 -4.59067 60.38396

C153 13.39637 -7.12819 60.88539

H154 12.58016 -7.83272 61.02631

C155 14.88792 -5.92187 59.41446

H156 15.22475 -5.68379 58.40934

C157 13.83995 -6.83066 59.59132

H158 13.36608 -7.28788 58.72625

H159 9.00376 0.70507 60.77526

C160 8.12698 0.15609 60.46501

H161 7.28367 0.45925 61.06777

O162 11.39757 -3.19338 64.11657

H163 12.22174 -2.78024 64.43265

H164 10.78525 -6.15878 62.84544

C165 10.41108 -7.00608 63.40104

H166 10.10408 -7.79168 62.7023

H167 11.27928 -7.41665 63.94093

N168 9.3717 -6.64113 64.35793

H169 9.3387 -5.65143 64.66165

C170 9.0717 -7.50467 65.35722

N171 9.27874 -8.81335 65.23436

H172 9.31834 -9.40346 66.10383

H173 9.51837 -9.2302 64.34269

N174 8.50998 -7.04561 66.4946

H175 8.7631 -6.10158 66.77418

H176 8.22993 -7.75388 67.20338

O177 6.75849 -3.48677 65.67449

H178 6.4819 -3.28046 64.76577

H179 6.55647 -4.43452 65.77399

O180 9.45333 -4.2273 65.74581

H181 8.62453 -3.70373 65.67653

H182 10.15901 -3.73851 65.24447

C183 8.62202 -12.88898 68.81297

H184 9.43595 -12.55922 69.47383

H185 8.04173 -13.63799 69.35894

C186 9.26041 -13.64954 67.62997

O187 9.26052 -14.88448 67.58325

C188 7.70165 -11.67688 68.48934

H189 7.0925 -11.45319 69.37254

H190 6.98771 -11.97074 67.70649

C191 8.30418 -10.3242 68.02092

O192 9.46798 -10.32272 67.47321

O193 7.58485 -9.30409 68.14547

N194 9.84063 -12.85717 66.68827

H195 9.86082 -11.84395 66.90845

C196 10.61799 -13.38699 65.58302

H197 10.1228 -14.28715 65.20758

H198 11.62395 -13.68307 65.91818

C199 10.76343 -12.35591 64.46236

H200 11.54875 -12.68651 63.76819

H201 11.08468 -11.38305 64.84891

C202 9.50873 -12.1912 63.63129

O203 8.66862 -13.0354 63.41313

O204 9.44003 -10.94109 63.06918

H205 11.11776 -2.72793 63.28113

H206 12.54338 -5.1018 57.706

C207 27.69198 -4.71607 52.704

H208 28.33184 -4.08171 52.08106

C209 27.30879 -3.97009 53.99664

H210 26.7324 -4.65265 54.63392

H211 28.24125 -3.74334 54.52485

C212 26.49723 -2.69588 53.72671

H213 27.07641 -2.03367 53.0691

H214 25.58819 -2.94903 53.16883

C215 26.11921 -1.89432 54.98647

H216 25.57362 -0.99346 54.67699

H217 27.03114 -1.5519 55.49525

C218 25.27518 -2.62903 56.03666

H219 25.81842 -3.47735 56.46469

H220 25.01792 -1.94953 56.85199

N221 23.99558 -3.1515 55.48342

H222 23.27703 -3.38158 56.20557

H223 23.48202 -2.45519 54.86264

H224 24.13891 -4.01865 54.90784

C225 24.52296 -7.55904 51.273

H226 23.62276 -7.93973 51.78081

O227 25.60579 -8.17021 51.31144

N228 24.31264 -6.39313 50.64628

H229 23.36007 -5.96177 50.74804

C230 25.36508 -5.75616 49.86593

H231 24.95454 -4.8255 49.46258

H232 25.66653 -6.39275 49.02833

C233 26.64593 -5.42598 50.62905

O234 27.72749 -5.39789 50.0361

N235 26.50252 -5.04491 51.9269

H236 25.62688 -5.21578 52.43967

O237 22.36855 -1.66816 53.87728

H238 21.57601 -1.61885 54.44495

H239 22.21232 -2.49322 53.32972

O240 21.4707 -3.68503 56.29178

H241 20.8246 -2.97274 56.50589

H242 21.0155 -4.53482 56.48526

O243 24.29917 -5.33051 53.76272

H244 24.0789 -6.24343 54.0427

H245 23.51362 -4.96733 53.22063

O246 23.12893 -2.78275 58.44538

C247 23.57791 -3.15413 59.50989

H248 24.50355 -3.70669 59.57568

H249 23.1142 -2.87673 60.44513

C250 28.50999 -5.95806 53.05401

O251 29.5343 -5.83138 53.73873

N252 28.01907 -7.16947 52.67977

H253 27.2021 -7.24214 52.07205

C254 28.53502 -8.42395 53.20699

H255 29.23255 -8.16523 54.00961

H256 29.09189 -8.9725 52.43587

C257 27.35781 -9.29385 53.69663

H258 27.73393 -10.23973 54.10235

H259 26.73106 -9.52651 52.83082

C260 26.49861 -8.55629 54.75699

H261 26.53007 -7.48005 54.57829

H262 26.88918 -8.74943 55.76408

C263 25.04395 -8.95565 54.73217

O264 24.87044 -10.28347 54.93219

O265 24.08497 -8.2121 54.56389

H266 23.90574 -10.44236 54.87897

H267 14.39819 -9.25167 56.55424

H268 13.2704 -6.82917 56.25294

H269 18.25928 -9.49876 50.15049

C270 17.58417 -8.6559 50.15203

H271 17.27723 -8.44202 49.13883

H272 13.57433 -8.8446 53.08581

**The TPSSh optimized structure (C, H2), protons are on the S2B and S5A**

Mo1 15.95485 -5.9927 53.41039

Fe2 8.72989 -7.2873 54.33703

Fe3 11.04575 -5.79416 55.12835

Fe4 10.98621 -6.88253 52.61891

Fe5 11.43472 -8.40909 54.73955

Fe6 14.08613 -7.97238 54.22631

Fe7 13.72726 -5.61555 55.21454

Fe8 13.40235 -5.91067 52.56396

C9 17.65272 -2.41818 54.40271

H10 17.94977 -1.48323 53.92595

H11 16.56602 -2.41155 54.54911

C12 18.0176 -3.6117 53.48671

C13 19.53876 -3.60848 53.18062

H14 20.06735 -3.91556 54.08909

H15 19.8252 -2.57227 52.96972

C16 19.9366 -4.50561 52.00635

H17 19.62398 -4.05079 51.05815

H18 19.39871 -5.45301 52.07069

C19 17.25457 -3.64996 52.14049

O20 16.78772 -4.83609 51.82659

O21 17.22092 -2.65347 51.42135

O22 17.64715 -4.83057 54.19208

C23 12.40819 -6.75666 53.99933

S24 10.07042 -7.4604 56.37752

S25 15.62774 -6.88703 55.65334

S26 9.40025 -5.19952 53.434

S27 12.25869 -4.27451 56.57823

S28 13.1414 -9.80044 55.15255

S29 14.45792 -4.09751 53.55791

S30 9.94616 -8.94752 52.95849

S31 14.88034 -7.6118 52.03849

S32 11.91501 -5.94625 50.58854

H33 13.63522 -2.19018 49.04254

C34 13.29789 -2.02415 50.05501

H35 13.84035 -2.71595 50.7043

H36 13.50271 -0.99318 50.36137

N37 11.86021 -2.31611 50.07826

H38 11.33646 -2.11651 49.23736

C39 11.16126 -2.90506 51.06466

N40 11.65739 -3.08554 52.28241

H41 11.14943 -3.7411 52.89815

H42 12.65717 -3.00243 52.47329

N43 9.85617 -3.22049 50.83836

H44 9.65705 -3.56943 49.90704

H45 9.44227 -3.81908 51.5742

H46 7.93851 0.29617 59.39829

C47 8.33262 -1.33658 60.8752

H48 8.24169 -1.38631 61.96788

H49 7.49949 -1.92637 60.46966

C50 9.63084 -2.01359 60.52499

N51 10.59255 -2.33937 61.48048

C52 10.03709 -2.48754 59.29696

H53 9.57275 -2.4773 58.32258

C54 11.54338 -2.99118 60.81759

H55 12.43971 -3.41221 61.24991

N56 11.2509 -3.09714 59.50118

H57 18.7327 -4.37995 57.65

H58 16.6959 -8.93115 50.72841

C59 18.24424 -7.42545 50.78446

H60 17.56486 -6.56981 50.74105

H61 19.15029 -7.15556 50.22831

C62 18.62922 -7.66018 52.21295

N63 17.79817 -7.37762 53.29326

C64 19.8156 -8.15941 52.69387

H65 20.72763 -8.43184 52.18789

C66 18.47335 -7.69616 54.38995

H67 18.10766 -7.57973 55.39811

N68 19.69576 -8.17993 54.06991

H69 20.42573 -8.4018 54.73289

H70 6.39285 -9.48834 56.52589

C71 6.77088 -9.54323 55.49979

H72 7.82777 -9.82359 55.52858

H73 6.21666 -10.3125 54.95188

S74 6.55026 -7.91116 54.66707

H75 11.78654 -3.57746 58.77157

H76 17.68615 -4.60059 55.18441

H77 8.64622 -10.95186 62.49559

C78 18.32606 -2.55255 55.77846

O79 18.11451 -3.67771 56.37803

O80 19.03443 -1.62547 56.24491

C81 21.41312 -4.87444 51.88054

O82 21.69779 -5.76721 51.03749

O83 22.27506 -4.28638 52.63193

H84 15.70246 -0.19964 61.49124

C85 14.855 -0.08296 60.83197

H86 13.9165 -0.28675 61.32611

H87 14.83347 0.95723 60.47828

C88 15.00915 -1.05754 59.65067

H89 14.22069 -0.86845 58.91064

H90 14.86266 -2.0809 60.01363

C91 16.36899 -0.99326 58.9462

H92 16.62894 0.04021 58.67648

H93 16.32826 -1.55089 58.00101

C94 17.50926 -1.58419 59.78157

O95 17.34396 -2.03675 60.91979

N96 18.73669 -1.5468 59.18344

H97 19.44708 -2.10291 59.64562

H98 18.82496 -1.47599 58.16241

C99 10.65202 0.00991 53.346

H100 11.16369 0.9593 53.28721

H101 9.58634 0.18507 53.35799

H102 10.91869 -0.58791 52.47228

C103 11.09596 -0.64904 54.66743

H104 10.802 -1.70654 54.65906

C105 10.39805 0.02597 55.85892

H106 9.30756 -0.07295 55.78586

H107 10.63737 1.09833 55.89598

H108 10.71484 -0.41929 56.80947

C109 12.62178 -0.55506 54.82356

H110 13.1511 -1.05916 54.00724

H111 12.95976 -1.00552 55.76461

H112 12.93956 0.49739 54.82991

H113 13.45501 -12.23047 53.18831

C114 13.91107 -11.62096 52.42198

H115 14.73666 -11.05163 52.86087

H116 14.25322 -12.24123 51.58814

N117 12.84924 -10.73035 51.95249

H118 12.20774 -10.3694 52.67338

C119 12.82522 -10.13585 50.77073

N120 13.90102 -10.25883 49.93214

H121 13.82224 -9.74243 49.06348

H122 14.79996 -10.11111 50.38438

N123 11.70358 -9.53168 50.33247

H124 10.95448 -9.41702 51.04327

H125 11.8245 -8.68823 49.77937

H126 19.20242 -8.21805 61.71256

C127 19.59792 -7.22989 61.99007

H128 20.63755 -7.36165 62.30511

C129 18.82186 -6.67962 63.18111

O130 19.36491 -6.22973 64.19085

C131 19.49183 -6.27179 60.79903

H132 19.91959 -5.30399 61.09186

H133 18.43849 -6.06883 60.5835

C134 20.14696 -6.73798 59.49308

H135 19.7001 -7.68912 59.16813

H136 21.22149 -6.92256 59.60468

C137 19.97248 -5.75464 58.33378

O138 20.73929 -5.74895 57.36724

O139 18.93231 -4.95369 58.48141

N140 17.46971 -6.70905 62.99646

H141 17.10821 -7.0201 62.10122

C142 16.53689 -6.00589 63.85304

H143 15.85699 -6.72723 64.32766

H144 17.13317 -5.53632 64.63951

C145 15.71631 -4.95641 63.0706

H146 16.37456 -4.14165 62.74929

H147 14.97629 -4.53009 63.76318

C148 15.01951 -5.55092 61.85915

C149 13.99082 -6.49863 62.00438

H150 13.66328 -6.78437 63.00318

C151 15.42873 -5.20357 60.56064

H152 16.21881 -4.46693 60.43413

C153 13.38614 -7.07723 60.88438

H154 12.59376 -7.8109 61.01232

C155 14.8231 -5.78152 59.43752

H156 15.14867 -5.50568 58.43872

C157 13.79921 -6.72094 59.59503

H158 13.33104 -7.16903 58.72281

H159 9.00363 0.70512 60.77548

C160 8.12722 0.15597 60.46464

H161 7.28365 0.45923 61.0679

O162 11.37515 -3.17455 64.15565

H163 12.22171 -2.78015 64.43269

H164 10.78522 -6.15879 62.84544

C165 10.41113 -7.00607 63.40104

H166 10.10407 -7.7917 62.70228

H167 11.27985 -7.41714 63.93967

N168 9.37251 -6.64216 64.35945

H169 9.33987 -5.65367 64.66554

C170 9.07568 -7.50645 65.35989

N171 9.28222 -8.8149 65.23591

H172 9.32235 -9.40541 66.10528

H173 9.51443 -9.23445 64.34307

N174 8.5199 -7.047 66.4998

H175 8.77241 -6.10129 66.77541

H176 8.24334 -7.75482 67.21036

O177 6.74765 -3.49501 65.67582

H178 6.48191 -3.28046 64.76577

H179 6.54652 -4.4441 65.76391

O180 9.44086 -4.23845 65.77417

H181 8.61271 -3.7145 65.6996

H182 10.15172 -3.74326 65.28726

C183 8.622 -12.88899 68.81301

H184 9.43843 -12.56147 69.47215

H185 8.04278 -13.63809 69.36013

C186 9.25527 -13.64944 67.62728

O187 9.25308 -14.88438 67.5801

C188 7.7036 -11.6743 68.49436

H189 7.09916 -11.44896 69.3804

H190 6.98535 -11.96577 67.71461

C191 8.30863 -10.32265 68.02499

O192 9.47172 -10.32343 67.47565

O193 7.5919 -9.30098 68.1523

N194 9.83616 -12.85748 66.68572

H195 9.86186 -11.84493 66.90861

C196 10.61801 -13.38699 65.583

H197 10.12546 -14.28784 65.20606

H198 11.62298 -13.68108 65.92278

C199 10.76627 -12.35581 64.46288

H200 11.55094 -12.68645 63.76818

H201 11.08856 -11.38352 64.85038

C202 9.5116 -12.19158 63.63167

O203 8.67256 -13.03663 63.41303

O204 9.4434 -10.94277 63.06557

H205 11.07583 -2.71196 63.32494

H206 12.53303 -3.14358 55.89921

C207 27.69197 -4.71604 52.70398

H208 28.33046 -4.08628 52.07514

C209 27.31239 -3.95993 53.99014

H210 26.74109 -4.6372 54.63739

H211 28.24595 -3.72211 54.51167

C212 26.4936 -2.6943 53.70395

H213 27.0623 -2.0432 53.02654

H214 25.57962 -2.96359 53.16187

C215 26.12501 -1.87114 54.95096

H216 25.56456 -0.98254 54.63298

H217 27.03969 -1.50912 55.44078

C218 25.30624 -2.5965 56.02579

H219 25.86898 -3.42499 56.46696

H220 25.04693 -1.9023 56.82816

N221 24.02477 -3.1519 55.50289

H222 23.31378 -3.27406 56.24944

H223 23.51592 -2.51303 54.80741

H224 24.15559 -4.06653 54.999

C225 24.52299 -7.55906 51.273

H226 23.61916 -7.94547 51.76958

O227 25.60983 -8.16319 51.32006

N228 24.31262 -6.39339 50.64642

H229 23.34158 -6.00023 50.72134

C230 25.36493 -5.75602 49.86601

H231 24.95592 -4.82414 49.46388

H232 25.66651 -6.39114 49.02706

C233 26.64604 -5.4261 50.62901

O234 27.72694 -5.3913 50.03636

N235 26.501 -5.04693 51.92885

H236 25.63052 -5.23163 52.44391

O237 22.31985 -1.8932 53.8556

H238 21.65017 -2.10729 54.5408

H239 22.2285 -2.68734 53.25282

O240 21.33232 -3.2279 56.22066

H241 20.60877 -2.6125 56.48747

H242 21.00789 -4.1248 56.46812

O243 24.30786 -5.37985 53.83837

H244 24.14122 -6.29901 54.13684

H245 23.50688 -5.08745 53.27859

O246 23.12485 -2.78987 58.44456

C247 23.57801 -3.15396 59.50998

H248 24.50353 -3.70676 59.57565

H249 23.11413 -2.87681 60.44509

C250 28.50999 -5.95808 53.05403

O251 29.54075 -5.82962 53.72864

N252 28.01117 -7.16904 52.69002

H253 27.18793 -7.24274 52.09023

C254 28.53502 -8.42395 53.20699

H255 29.26827 -8.16662 53.97747

H256 29.05155 -8.98433 52.41665

C257 27.37067 -9.27451 53.75388

H258 27.74583 -10.235 54.12478

H259 26.68832 -9.47767 52.92233

C260 26.59867 -8.53047 54.87442

H261 26.61311 -7.45662 54.67919

H262 27.06925 -8.71606 55.84743

C263 25.14992 -8.93888 54.95154

O264 24.99972 -10.24228 55.29082

O265 24.17764 -8.22731 54.73529

H266 24.03562 -10.41287 55.28546

H267 12.83959 -6.77856 50.05257

H268 18.2594 -9.49869 50.15035

C269 17.58353 -8.65594 50.15247

H270 17.27761 -8.44203 49.1386

**The TPSSh optimized structure (C, H2),protons are on the S2B and S3A**

Mo1 16.02778 -5.92719 53.4126

Fe2 8.90865 -7.3065 54.53324

Fe3 11.13523 -5.62928 55.03975

Fe4 10.94487 -6.7587 52.60393

Fe5 11.54161 -8.2608 54.79891

Fe6 14.20464 -7.83465 54.17185

Fe7 13.80857 -5.50035 55.09355

Fe8 13.44372 -5.9179 52.46883

C9 17.74371 -2.34688 54.4419

H10 18.03942 -1.41093 53.96596

H11 16.66263 -2.33662 54.62175

C12 18.07937 -3.53419 53.50783

C13 19.59403 -3.53473 53.18015

H14 20.1491 -3.82006 54.08095

H15 19.8613 -2.49963 52.93779

C16 19.97263 -4.44467 52.01139

H17 19.63329 -4.00833 51.06378

H18 19.45022 -5.39873 52.10027

C19 17.31851 -3.56854 52.16034

O20 16.88251 -4.76356 51.83954

O21 17.28094 -2.56934 51.44604

O22 17.70481 -4.75223 54.21175

C23 12.506 -6.71232 54.00944

S24 10.25772 -7.37303 56.49548

S25 15.71389 -6.77952 55.65766

S26 9.32274 -5.18458 53.59551

S27 12.41224 -4.42729 56.71321

S28 13.36302 -9.83059 55.25438

S29 14.52136 -4.04126 53.45578

S30 10.04974 -8.88449 53.05632

S31 15.00633 -7.53084 52.01875

S32 11.99052 -5.99105 50.69487

H33 13.63514 -2.19022 49.04252

C34 13.29796 -2.02411 50.05508

H35 13.86528 -2.68814 50.71254

H36 13.45435 -0.98098 50.34752

N37 11.87129 -2.37614 50.06489

H38 11.37663 -2.30194 49.18671

C39 11.17618 -2.95071 51.05902

N40 11.63679 -3.00563 52.30403

H41 11.17335 -3.6796 52.92612

H42 12.63284 -2.89278 52.48959

N43 9.89207 -3.3413 50.80796

H44 9.79665 -3.83628 49.92573

H45 9.49875 -3.91347 51.56934

H46 7.93769 0.29788 59.39884

C47 8.35957 -1.33286 60.86664

H48 8.27491 -1.39732 61.95888

H49 7.54601 -1.94544 60.45522

C50 9.68397 -1.94088 60.49533

N51 10.65333 -2.28737 61.43509

C52 10.12946 -2.28252 59.23841

H53 9.67362 -2.21799 58.26255

C54 11.6483 -2.82256 60.73363

H55 12.56213 -3.22841 61.14315

N56 11.37705 -2.82963 59.40785

H57 18.78425 -4.34765 57.65386

H58 16.70258 -8.92191 50.73857

C59 18.25866 -7.42073 50.76718

H60 17.58201 -6.56237 50.72266

H61 19.15228 -7.15959 50.18709

C62 18.68031 -7.6192 52.19259

N63 17.88484 -7.29138 53.28826

C64 19.88787 -8.08306 52.65738

H65 20.78691 -8.36689 52.13482

C66 18.60232 -7.54494 54.37444

H67 18.27726 -7.37373 55.38797

N68 19.81715 -8.03413 54.03668

H69 20.57501 -8.19614 54.68662

H70 6.39283 -9.48836 56.5259

C71 6.82919 -9.52209 55.52439

H72 7.87461 -9.83419 55.60486

H73 6.28803 -10.25496 54.91666

S74 6.69543 -7.85455 54.75386

H75 11.94031 -3.22371 58.64735

H76 17.70515 -4.52637 55.20226

H77 8.6413 -10.9469 62.50401

C78 18.4763 -2.53451 55.78042

O79 18.13999 -3.60451 56.4237

O80 19.35473 -1.72308 56.16955

C81 21.44975 -4.78583 51.86455

O82 21.73402 -5.70067 51.04573

O83 22.31541 -4.15593 52.57802

H84 15.7025 -0.19963 61.49121

C85 14.85496 -0.08303 60.83205

H86 13.91649 -0.28671 61.32608

H87 14.8331 0.95937 60.48481

C88 15.01661 -1.04731 59.64171

H89 14.22963 -0.85485 58.9017

H90 14.87499 -2.07484 59.99469

C91 16.37851 -0.97635 58.9372

H92 16.648 0.06157 58.69475

H93 16.32916 -1.51027 57.9786

C94 17.51255 -1.60323 59.75407

O95 17.33767 -2.09016 60.87602

N96 18.74921 -1.56131 59.17028

H97 19.44167 -2.1509 59.61832

H98 18.86568 -1.46308 58.15811

C99 10.65202 0.01 53.34621

H100 11.16369 0.95929 53.28718

H101 9.58632 0.18501 53.35785

H102 10.91615 -0.58765 52.47205

C103 11.07668 -0.66781 54.66446

H104 10.7384 -1.71195 54.65284

C105 10.40701 0.033 55.8574

H106 9.31313 -0.03666 55.79679

H107 10.67485 1.09905 55.88331

H108 10.72382 -0.41141 56.80796

C109 12.60316 -0.65084 54.83247

H110 13.11589 -1.101 53.97568

H111 12.91017 -1.20783 55.72466

H112 12.96554 0.38269 54.93158

H113 13.45504 -12.23043 53.18826

C114 13.91104 -11.62099 52.42204

H115 14.75799 -11.07927 52.85791

H116 14.2449 -12.25285 51.5929

N117 12.87716 -10.69948 51.94418

H118 12.12509 -10.42675 52.58531

C119 12.98328 -9.95352 50.84225

N120 14.11059 -10.04431 50.07945

H121 14.15331 -9.35455 49.336

H122 14.98029 -10.07927 50.6029

N123 11.95388 -9.21415 50.40328

H124 11.13523 -9.20533 51.02838

H125 12.16924 -8.23715 50.12106

H126 19.18458 -8.21191 61.7166

C127 19.59788 -7.22991 61.99003

H128 20.63808 -7.37671 62.29551

C129 18.8314 -6.66612 63.1826

O130 19.37838 -6.17471 64.16987

C131 19.4919 -6.27177 60.79908

H132 20.01421 -5.33887 61.04411

H133 18.44572 -5.98897 60.64673

C134 20.0216 -6.82836 59.47378

H135 19.50101 -7.7638 59.22265

H136 21.0877 -7.08082 59.52444

C137 19.83567 -5.90166 58.27415

O138 20.41936 -6.11298 57.20668

O139 19.00912 -4.89714 58.49941

N140 17.47556 -6.73156 63.01958

H141 17.11381 -7.07477 62.13623

C142 16.53688 -6.00588 63.85304

H143 15.85986 -6.71626 64.34724

H144 17.13013 -5.51082 64.62621

C145 15.71249 -4.98304 63.04028

H146 16.36478 -4.17304 62.69598

H147 14.9675 -4.54281 63.7186

C148 15.02281 -5.62265 61.84874

C149 14.02043 -6.592 62.02956

H150 13.70517 -6.85408 63.03854

C151 15.41462 -5.30084 60.5384

H152 16.18153 -4.54494 60.38528

C153 13.42611 -7.2205 60.93221

H154 12.65127 -7.96767 61.08681

C155 14.81963 -5.92781 59.43597

H156 15.12406 -5.66791 58.42576

C157 13.82534 -6.89122 59.63178

H158 13.35989 -7.37445 58.77694

H159 9.00377 0.70505 60.77522

C160 8.1269 0.15615 60.46486

H161 7.28372 0.45922 61.0679

O162 11.39718 -3.19472 64.11996

H163 12.22175 -2.78024 64.43267

H164 10.78534 -6.15881 62.8455

C165 10.41095 -7.00603 63.40099

H166 10.10411 -7.79171 62.70229

H167 11.27951 -7.4169 63.93999

N168 9.37263 -6.64167 64.35946

H169 9.34088 -5.65193 64.66383

C170 9.07784 -7.50443 65.36152

N171 9.28534 -8.81299 65.23957

H172 9.3282 -9.40259 66.10962

H173 9.51626 -9.23322 64.34687

N174 8.52284 -7.044 66.50161

H175 8.77606 -6.09873 66.77725

H176 8.24777 -7.75159 67.21323

O177 6.76018 -3.49141 65.67287

H178 6.48191 -3.28047 64.76577

H179 6.55939 -4.43988 65.76786

O180 9.45428 -4.22913 65.74846

H181 8.6254 -3.7058 65.67734

H182 10.16084 -3.74052 65.2479

C183 8.62205 -12.88898 68.81294

H184 9.43891 -12.56539 69.47343

H185 8.04004 -13.63743 69.35788

C186 9.25399 -13.64866 67.626

O187 9.24782 -14.88343 67.57568

C188 7.70736 -11.67085 68.49599

H189 7.10428 -11.44463 69.38268

H190 6.98755 -11.9596 67.71664

C191 8.31542 -10.32027 68.02709

O192 9.47864 -10.32293 67.47773

O193 7.59969 -9.29764 68.1517

N194 9.83707 -12.85655 66.68573

H195 9.86597 -11.84441 66.91064

C196 10.61797 -13.38699 65.58305

H197 10.12445 -14.28762 65.20688

H198 11.62303 -13.68186 65.92198

C199 10.76655 -12.35663 64.4621

H200 11.54897 -12.69015 63.76619

H201 11.09196 -11.38499 64.84848

C202 9.51037 -12.18967 63.63368

O203 8.67007 -13.03341 63.4151

O204 9.4429 -10.94051 63.06779

H205 11.11187 -2.72745 63.28687

H206 12.36035 -5.42005 57.62755

C207 27.69199 -4.71607 52.704

H208 28.33148 -4.08077 52.08173

C209 27.30265 -3.9706 53.99556

H210 26.72759 -4.65522 54.63202

H211 28.23194 -3.73766 54.5267

C212 26.4846 -2.7016 53.71916

H213 27.06651 -2.03481 53.06855

H214 25.58432 -2.96102 53.15

C215 26.08457 -1.90236 54.97344

H216 25.54473 -1.00115 54.65472

H217 26.98728 -1.55945 55.49824

C218 25.22219 -2.63579 56.00951

H219 25.75795 -3.4828 56.44937

H220 24.9507 -1.95508 56.81892

N221 23.95254 -3.16202 55.43603

H222 23.2144 -3.37622 56.14492

H223 23.45491 -2.47776 54.79024

H224 24.10668 -4.03958 54.87904

C225 24.52298 -7.55907 51.273

H226 23.6195 -7.94216 51.77283

O227 25.60849 -8.16564 51.32372

N228 24.31344 -6.39671 50.64179

H229 23.34856 -5.98838 50.7266

C230 25.365 -5.75607 49.866

H231 24.95439 -4.82383 49.46611

H232 25.66834 -6.38774 49.02504

C233 26.64597 -5.42605 50.62901

O234 27.72703 -5.39474 50.03541

N235 26.50251 -5.04589 51.9271

H236 25.629 -5.22537 52.44066

O237 22.33629 -1.73171 53.77156

H238 21.54133 -1.68192 54.33556

H239 22.19004 -2.56909 53.23981

O240 21.42393 -3.66441 56.27034

H241 20.75486 -2.96554 56.45689

H242 20.99182 -4.52178 56.48374

O243 24.29333 -5.36366 53.75598

H244 24.09138 -6.27882 54.0434

H245 23.50838 -5.02766 53.19759

O246 23.12536 -2.78786 58.44532

C247 23.57802 -3.154 59.51

H248 24.50352 -3.70674 59.57564

H249 23.11414 -2.8768 60.44508

C250 28.50999 -5.95805 53.054

O251 29.53746 -5.8309 53.73393

N252 28.01383 -7.16919 52.68633

H253 27.1948 -7.24267 52.0809

C254 28.53502 -8.42396 53.207

H255 29.2418 -8.16637 54.00189

H256 29.08266 -8.97195 52.4289

C257 27.36382 -9.29359 53.70931

H258 27.74241 -10.24504 54.09958

H259 26.72061 -9.5143 52.85224

C260 26.52834 -8.56172 54.79206

H261 26.55659 -7.48479 54.61673

H262 26.93968 -8.7615 55.78929

C263 25.07463 -8.96317 54.78851

O264 24.90548 -10.28619 55.0275

O265 24.11313 -8.22892 54.60002

H266 23.94094 -10.44766 54.98012

H267 13.53036 -9.37328 56.51231

H268 18.25926 -9.49878 50.15053

C269 17.58416 -8.65588 50.15195

H270 17.27723 -8.44203 49.13885
